# A programmable fungal platform for engineered living textiles

**DOI:** 10.1126/sciadv.aed6937

**Published:** 2026-07-24

**Authors:** Ke Li, Bolin An, Jicong Zhang, Dongmin Xun, Xinyi Yuan, Youyang Song, Xinyu Wang, Chao Zhong

**Affiliations:** ^1^ State Key Laboratory of Quantitative Synthetic Biology, Shenzhen Key Laboratory of Materials Synthetic Biology, Shenzhen Institute of Synthetic Biology, Shenzhen Institutes of Advanced Technology, Chinese Academy of Sciences, Shenzhen 518055, China.; ^2^ PEELSPHERE GMBH, Berlin, Germany.

## Abstract

A central challenge in engineered living materials (ELMs) is the seamless integration of macroscopic structural assembly with sustained cellular viability and programmable function. Here, we report a fungal-based living material that addresses this challenge by preserving the metabolic activity of *Cordyceps militaris* mycelia within macroscale, cohesive films fabricated via a low-energy process. These living textiles retain the capacity for environmental response, demonstrated by nutrient-induced aerial hyphal growth that enables surface renewal. The native mycelial architecture further allows for volumetric integration of engineered microbial partners, exemplified by coculture with pigment-producing *Saccharomyces cerevisiae* for in situ patterning and melanized *Aspergillus niger* for built-in ultraviolet shielding. This modular design decouples bulk structural fabrication from genetic functionalization, offering a plug-and-play platform for synthetic biology. Environmental assessments confirm near-complete morphological degradation within 41 days. Our work establishes a scalable and sustainable chassis for functional ELMs, bridging a critical gap between structural integrity and biological programmability.

## Teaser

A programmable fungal platform enables engineered living textiles with tunable functions and biodegradability.

## INTRODUCTION

The pursuit of sustainable materials with adaptive and regenerative capabilities has motivated the development of engineered living materials (ELMs), composite systems that harness metabolically active cells to achieve self-repair, environmental responsiveness, and programmable biochemical functions (*1*–*10*). These dynamic properties, unattainable in conventional static biomaterials, position ELMs as a promising paradigm for next-generation material design (*4*, *11*–*17*). While synthetic biology has greatly expanded the functional capabilities of ELMs (*12*, *15*, *18*), a persistent challenge lies in integrating autonomous structural assembly with sustained biological activity at macroscopic scales (*19*). Achieving such integration is essential for practical applications, from adaptive textiles to architectural biomaterials, where mechanical robustness, spatial uniformity, and scalable fabrication must converge with engineered biological function.

Current ELM platforms each present distinct limitations. Engineered bacterial biofilms (e.g., from *Escherichia coli*, *Bacillus subtilis*, or *Corynebacterium glutamicum*) enable genetic programmability and self-organization but typically yield only microscale or mechanically fragile assemblies (*1*–*3*, *20*–*27*). Bacterial cellulose, including that produced in kombucha, forms robust macroscale structures yet consists predominantly of acellular polysaccharide with limited embedded metabolic activity, constraining its dynamic functionality (*28*–*31*). Incorporation of engineered yeast has introduced sensing capabilities, although cells often remain confined to surface layers, precluding volumetric integration (*11*, *32*). Similarly, protein-based materials such as *Caulobacter crescentus* S-layers exhibit precise nanoscale ordering and can be fabricated into freestanding films, but they lack embedded cellular viability and metabolic adaptability (*19*, *24*).

In contrast, filamentous fungi have innate traits conducive to macroscale ELM construction: Their mycelial networks are entirely living, grow directionally from localized nutrient sources, and self-assemble into continuous, three-dimensional architectures without exogenous scaffolding (*33*–*39*). These traits have enabled widespread use of fungal-derived materials in packaging, architecture, and textiles (*33*, *38*, *40*–*44*). However, existing mycelium-based material processes typically involve devitalization, through desiccation, heating, or chemical cross-linking, which abrogates cellular viability and severs the link between biological function and structural utility (*45*, *46*).

**Fig. 1.**
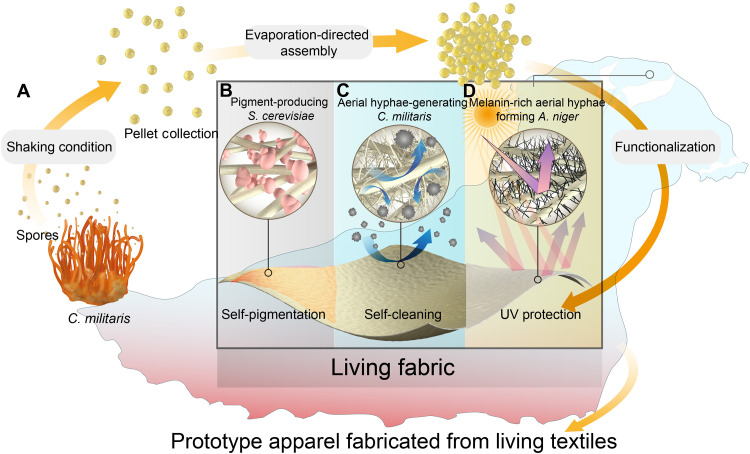
Biofabrication and multifunctional properties of engineered living textiles. The mycelial textile platform combines the structural integrity of *C. militaris* pellets with programmable metabolic functions from engineered microbes. (**A**) Schematic of the fabrication process showing pellet self-assembly into pellicles. The resulting living fabric exhibits distinct functionalities, including (**B**) self-pigmentation achieved via engineered yeast producing natural pigments; (**C**) self-cleaning properties provided by hydrophobic, structured aerial mycelia; and (**D**) intrinsic ultraviolet (UV) protection ascribed to melanin-rich aerial hyphae. This platform can be further tailored through additional functionalization strategies, enabling diverse applications ranging from sustainable textiles to advanced bioengineered materials. Illustration credit: L. Chen.

Here, we report a fabrication methodology that preserves mycelial viability while producing cohesive, flexible, and metabolically active films from *Cordyceps militaris*. Using pellet-based inoculation followed by low-energy fusion and glycerol plasticization, we generated dense hyphal networks that retain environmental responsiveness and regenerative capacity. As a proof-of-concept living textile, the material undergoes nutrient-induced aerial hyphal growth, enabling autonomous surface renewal without synthetic coatings (Fig. 1). Furthermore, the living fungal matrix supports modular and volumetric integration of engineered microbial partners without compromising structural integrity, circumventing the need for direct fungal genetic modification. We demonstrate this plug-and-play programmability through two complementary functional modules: (i) a chitin-binding, pigment-producing *Saccharomyces cerevisiae* strain for stable surface coloration, and (ii) melanized *Aspergillus niger* hyphae for integrated ultraviolet (UV) protection. Life cycle assessment (LCA; ISO 14040/14044) and biodegradation studies confirm the environmental compatibility of the material system. Together, this work establishes a scalable and functionally extensible platform for fungal-based ELMs, reembedding biological activity into structurally robust and environmentally responsive materials.

## RESULTS

### Viability-preserving, growth-enabled assembly of fungal pellicles

**Fig. 2.**
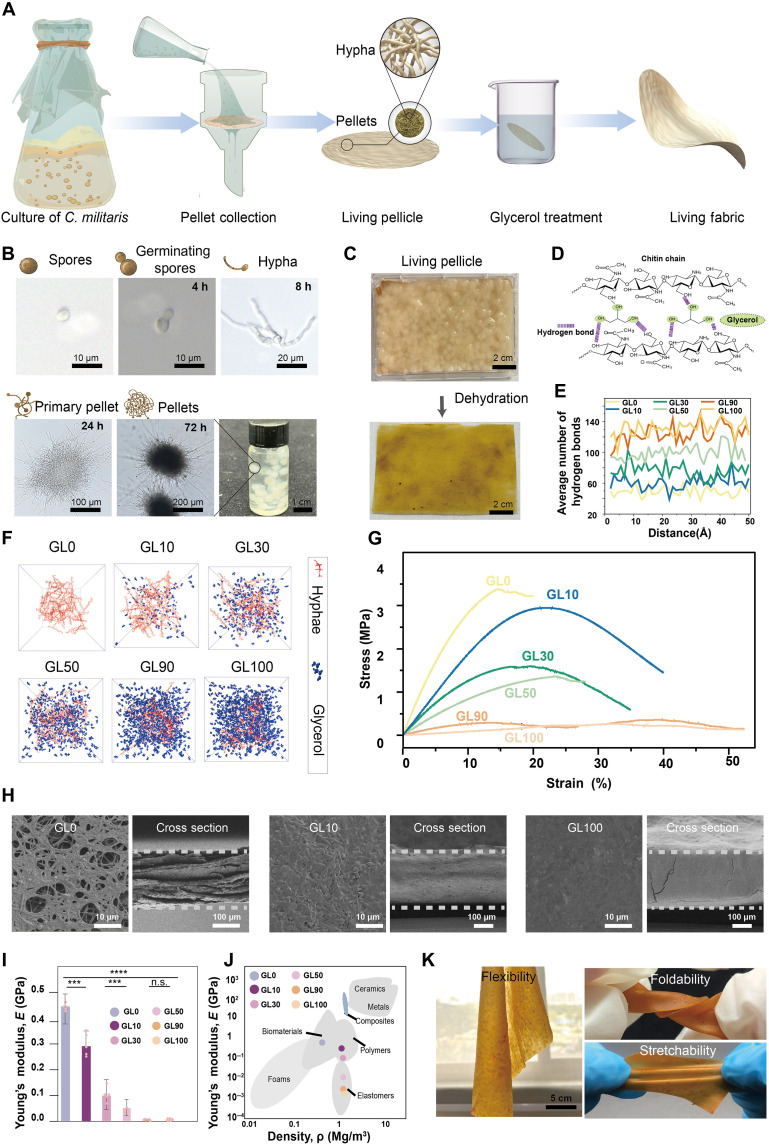
Fabrication workflow and material characterization of the living fungal textiles. (**A**) Schematic representation of the hyphal pellets culture and pellicle preparation process. *C. militaris* was precultured, collected as pellets, and processed into flexible pellicles, followed by treatment with glycerol. (**B**) Optical microscopy images show the growth of *C. militaris* under shaking conditions. Spores germinated within 4 hours and began to produce hyphae at 8 hours. After liquid culture for 24 hours, hyphal pellets formed in the liquid medium. h, hours. (**C**) Optical microscopy images of collected hyphal pellets before and after dehydration. (**D**) Schematic showing the potential hydrogen bonding networks formed between glycerol and chitin, the primary component of the hyphal cell wall. (**E**) Hydrogen bond count from MD simulations for GL0, GL10, GL30, GL50, GL90, and GL100, indicating the effect of the glycerol concentration on hydrogen bond formation. (**F**) MD simulation images showing the spatial distribution of glycerol molecules interacting with polysaccharides in hyphal pellicles at different glycerol concentrations. (**G**) Stress-strain curves from simulations, illustrating the variable mechanical properties of hyphal pellicles treated with varying glycerol concentrations. (**H**) SEM images of the surface and cross sections of GL0, GL10, and GL100-treated hyphal pellicles. (**I**) Young’s modulus measurements for hyphal pellicles treated with different glycerol concentrations. (**J**) Classification of hyphal pellicles based on mechanical properties and comparison with common materials. (**K**) Images of a GL10 fabric sample demonstrating its flexibility, foldability, and stretchability. Data are presented as means ± SD. Statistical significance was determined by two-tailed Student’s *t* test. n.s., not significant; ***P < 0.001; ****P < 0.0001. (A) Illustration credit: L. Chen.

Modular assembly using discrete bioactive building blocks is widely used in ELMs to enable controllable fabrication and functional integration, supporting reproducible handling and postfabrication reconfiguration (*4*, *29*). Mycelial pellets represent a naturally occurring embodiment of modular assembly, arising as a submerged morphological state of filamentous fungi and being highly tunable to environmental conditions (*47*). To fabricate freestanding living fungal pellicles, we first cultivated *C. militaris* spores under defined conditions to generate mycelial pellets as modular, bioactive building blocks (figs. S1 to S3). These pellets, rich in chitin and α/β-glucans, serve as compact units that can be molded into hyphal pellicles with geometries not achievable using flat mycelial mats (Fig. 2A) while maintaining comparable bulk mechanical performance (fig. S4).

To optimize pellet formation, we systematically varied spore concentrations (10^4^ to 10^8^ spores/ml), pH levels (*2*–*10*), and incubation temperatures (16° to 32°C; figs. S1 to S3). Optimal conditions, that is, 1 × 10^6^ spores/ml, pH 8, and 24°C, produced uniform fibrous pellets with high biomass yield and good molding potential (Fig. 2B). We then placed the purified pellets into molds of predefined shapes and dried them at 45°C for 6 hours. The inherent cohesive properties within the hyphal network enabled the dried pellets to consolidate gradually, yielding solid structures conforming precisely to the mold geometry (Fig. 2C). This simple drying-and-molding strategy highlights the versatility of *C. militaris* biomass, which can be shaped into both two- and three-dimensional constructs with minimal chemical intervention (fig. S5).

Furthermore, the pellet-based fabrication strategy also allows modular structural repair or extension after initial shaping (*29*). Dried fungal pellicles subjected to mechanical cuts or damage could be effectively repaired by applying fresh wet pellets to the damaged regions (fig. S6). Pellets with a hydration state comparable to the original matrix were placed to ensure intimate interfacial contact with the surrounding intact pellicle. Following drying, the newly integrated pellets bonded firmly to the preexisting structure via interfacial hydrogen bonding, effectively reestablishing material continuity. The applied pellet volume was adjusted according to defect size, maintaining an areal density consistent with that used during initial fabrication. This reassembly capability underscores the intrinsic modularity of fungal biomaterials, supporting postproduction repair and structural augmentation without the need for external adhesives or chemical processing.

A common limitation associated with biomass-derived materials is that they typically form dense hydrogen-bonding networks that restrict chain mobility and lead to brittle fracture under mechanical stress (*48*, *49*). Preliminary evaluations indicated that fungal films derived from *C. militaris* pellets could be readily formed, but their mechanical performance was unsatisfying, particularly in terms of flexibility and durability, compared with conventional polymer-derived textiles. To address the brittleness in these biomass-based materials, we introduced glycerol as a plasticizer, which is known to increase flexibility in polysaccharide-rich systems by disrupting interchain hydrogen bonding and reducing stiffness through its hygroscopic nature (Fig. 2A) (*48*–*50*).

To investigate the molecular effects of glycerol incorporation on both flexibility and structural strength, we first performed molecular dynamics (MD) simulations using the AMBER force fields, focusing on chitin- and glucan-rich cell wall components that typify *C. militaris*. We modeled glycerol concentrations at 0, 10, 30, 50, 90, and 100% (v/v), designated as GL0, GL10, GL30, GL50, GL90, and GL100, respectively (table S1 and fig. S7). The simulation results indicated that glycerol readily forms hydrogen bonds with polysaccharide chains, disrupting native intermolecular forces (Fig. 2, D and E). Under uniaxial tension, a progressive breakdown of hydrogen bonds correlated with enhanced elasticity and elongation (fig. S8). However, high glycerol contents (GL90 and GL100) led to partial phase separation (Fig. 2F and fig. S9), undermining the cohesive benefits of plasticization. Stress-strain analysis based on simulations further showed that moderate glycerol concentrations (10 to 50%) achieve the best compromise between flexibility and structural strength (Fig. 2G).

To experimentally validate the trends predicted by MD simulations, we prepared fungal pellicles treated with a range of glycerol concentrations. The goal was to determine whether changes in microstructure and mechanical properties would align with molecular-level predictions regarding hydrogen bond disruption and phase behavior. Dried *C. militaris* pellicles were subsequently immersed in glycerol solutions at 10, 30, and 50% (v/v) for 24 hours. For glycerol concentrations above 50%, direct incorporation into the wet fungal matrix was used instead of postdrying immersion to ensure homogeneous distribution and avoid surface-limited plasticization. After air-drying at ambient temperature, the as-prepared materials were examined for morphological and mechanical changes.

Scanning electron microscopy (SEM) revealed pronounced glycerol-dependent microstructural evolution within the fungal pellicles. In the absence of glycerol (GL0), the matrix exhibited a highly porous architecture accompanied by visible microcracks, indicative of intrinsic brittleness. Incorporation of glycerol at 10% (GL10) markedly suppressed crack formation and yielded a more continuous and cohesive surface morphology, consistent with enhanced plasticization. At elevated glycerol levels, the pellicles also appeared thicker and more swollen, with reduced stratification in cross-section. While large-scale cracking diminished, localized surface features reflected plasticizer-induced matrix expansion rather than structural densification alone (Fig. 2H and figs. S10 and S11). Nitrogen adsorption analysis further quantified this microstructural transition. The Brunauer-Emmett-Teller–specific surface area decreased progressively with increasing glycerol content, from ∼80 m^2^/g (GL0) to ∼2 to 3 m^2^/g (GL2.5), <1 m^2^/g (GL5), and near-baseline levels at GL10, indicating a continuous reduction in accessible pore networks rather than an abrupt structural collapse (fig. S12). These results underscore a key design insight: Flexibility can be enhanced through moderate glycerol addition, but maintaining durability requires avoiding phase separation, highlighting the importance of optimizing the plasticizer content to ensure mechanical reliability during repeated deformation (*10*, *50*).

Mechanical testing was used to quantify these structural differences. As the glycerol concentration increased from 0 to 100%, Young’s modulus decreased significantly from 0.5 ± 0.0012 GPa (control) to 0.0009 ± 0.0001 GPa (GL100), indicating notable stiffness reduction. Notably, the GL10 sample exhibited a 50 ± 8% improvement in ductility compared with the untreated film, demonstrating enhanced flexibility without loss of durability. At higher concentrations (>GL50), the pellicles approached elastomeric behavior, with soft, extensible properties but reduced shape retention and strength (Fig. 2, I and J, and fig. S13).

In addition to the mechanical performance, fungal-based materials need to maintain a reliable performance under environmental stress. Therefore, we systematically evaluated the surface wettability and thermal stability of glycerol-treated pellicles because these factors are critical for practical handling, postprocessing, and usage under varying ambient conditions. Water contact angle (WCA) measurements revealed that the glycerol-free pellicle had a WCA of 61° ± 2°. However, as the glycerol concentration increased, the WCA gradually decreased, reflecting the increasing hydrophilicity of the pellicles due to the hygroscopic nature of glycerol. Specifically, pellicles treated with 10% glycerol (GL10) exhibited a WCA of 51° ± 4°, whereas those treated with 30% (GL30), 50% (GL50), 90% (GL90), and 100% (GL100) glycerol displayed WCAs of 40° ± 3°, 33° ± 5°, 25° ± 6°, and 23° ± 3°, respectively (table S2). Thermogravimetric analysis and differential scanning calorimetry (DSC) indicated that hyphal-based pellets, whether glycerol treated or not, began to degrade above 200°C (fig. S14). The first ∼5% mass loss up to 200°C corresponded to the evaporation of loosely bound water, whereas the subsequent ∼80% loss between 200° and 375°C was ascribed to the breakdown of organic components such as amino acids, polysaccharides, and chitin. DSC results revealed similar glass transition temperatures (∼180°C) for all samples (fig. S15), suggesting that glycerol treatment did not fundamentally alter the thermal stability of the pellicles.

To further gauge the real-world feasibility of these glycerol-plasticized composites, we examined their chemical resilience. A GL10 cubic sample with dimensions of 2 cm by 2 cm by 0.2 cm was sequentially soaked in various solvents, including hexane, ethanol, acetonitrile, acetone, dimethyl sulfoxide, and chloroform each for 24 hours (fig. S16). After treatment, SEM data revealed no noticeable morphological changes relative to water-exposed controls. Attenuated total reflectance–Fourier transform infrared (ATR-FTIR) spectroscopy analysis similarly indicated no major chemical alterations (fig. S16). Thus, glycerol-plasticized hyphal pellicles exhibit a robust solvent tolerance, an attractive feature for many industrial processes.

To further evaluate the functional potential of glycerol-plasticized fungal pellicles, we investigated their shape adaptability, mechanical integrity under load, and reparability, three properties critical for practical deployment in dynamic or reusable applications. Based on earlier mechanical and structural data, GL10 was selected as a representative formulation due to its favorable balance between flexibility and durability. The pellicles could be readily folded, twisted, or shaped into complex geometries, such as origami cranes, without cracking (Fig. 2K and figs. S17 and S18). In addition, they demonstrated load-bearing capacity. Specifically, four 1-mm-wide, 0.18-cm-thick GL10 strips were twisted into a rope that successfully supported a 1-kg weight (fig. S19). These observations confirm that the partial plasticization caused by 10% glycerol enables sufficient molecular mobility to allow elastic bending, and the mechanical integrity is not sacrificed at the expense of flexibility. Furthermore, we evaluated the reparability of GL10 pellicles. Mimicking superficial damage, we introduced shallow cuts and complete incisions into the material. Upon application of a few microliters of water and subsequent ambient drying, the cuts visibly rejoined, restoring the material’s continuity (figs. S20 and S21). Mechanical testing further revealed that repaired samples retained robust load-bearing capacity across repeated repair cycles, maintaining ∼68% of the original stiffness even after 10 repair cycles (fig. S22). This water-assisted self-healing behavior likely results from the reversible hydrogen bonding and chain interdiffusion facilitated by glycerol. Such intrinsic repairability extends the material life span and aligns with circular material design principles.

These findings demonstrate that *C. militaris* hyphal pellicles plasticized with 10 to 50% glycerol provide a strong balance of mechanical strength, thermal stability, solvent resistance, and operational flexibility. The agreement between MD predictions and bench-scale experiments lays a rigorous foundation for further optimization and upscaling, designating *C. militaris* as a promising scaffold for sustainable polymer alternatives.

### Postgrowth pigmentation via engineered *S. cerevisiae* coculture

To enable biologically based coloration of mycelial textiles, we developed a strategy for postgrowth pigmentation using the engineered pigment-producing microbes. While *C. militaris* has endogenous pigment biosynthesis pathways, its limited genetic tractability and low pigment yield pose substantial barriers for tunable coloration (*51*). As an alternative, we introduced engineered *S. cerevisiae* as a modular pigmentation module, motivated by its biosafety profile, high pigment productivity, and well-established surface-display engineering toolkit for stable functional integration (*11*, *52*). Engineered yeast cells preproducing pigments were integrated onto preformed *C. militaris* mycelial pellets before textile consolidation, enabling postgrowth pigmentation through modular yeast-mycelium assembly.

**Fig. 3.**
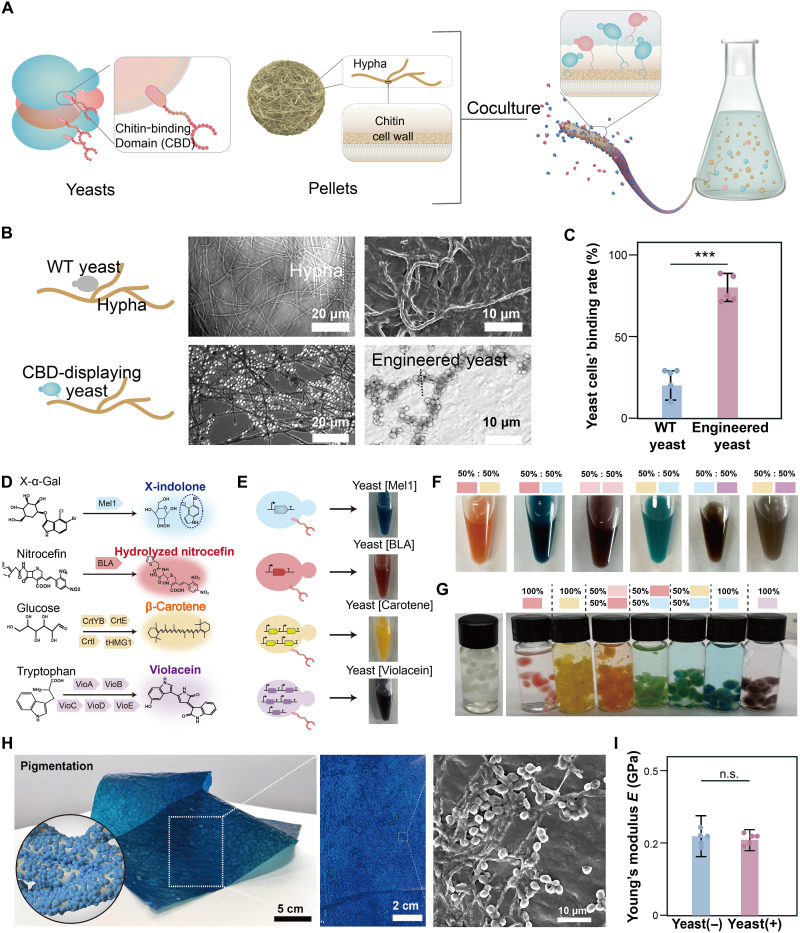
Autonomous pigmentation of mycelium textiles through cocultured engineered *S. cerevisiae*. (**A**) Schematic representation of the hyphal cell wall composition, primarily chitin, and the design of engineered yeasts displaying chitin-binding domains (CBDs) on cell surfaces that enhance binding between the hyphal surface and engineered yeasts through noncovalent interactions between chitin and CBD. (**B**) Microscopic (top) and SEM (bottom) images showing uniform binding of CBD-displaying yeasts along the hyphal surface, whereas yeasts without CBDs exhibit minimal attachment. WT, wild type. (**C**) Quantification of the yeast binding efficiency through optical density at 600 nm (OD_600_) measurements before and after 6 hours of ultrasonication, showing a 75 and 18% binding efficiency for CBD- and non-CBD displaying yeasts, respectively. (**D** and **E**) Engineered yeast strains producing distinct color outputs through enzymatic substrate conversion or de novo pigment biosynthesis, including α-galactosidase/Mel1-mediated blue coloration, β-lactamase-mediated red coloration, β-carotene biosynthesis, and violacein biosynthesis. (**F**) Pigmentation diversity achieved by mixing engineered yeast strains, generating a color palette tunable from red to purple. (**G**) Optical images of colored hyphal composites created by integrating pigment-producing yeasts into the hyphal matrix. (**H**) Optical image of a blue fabric formed by combining GL10 with engineered yeasts expressing the mel1 enzyme for pigment production. (**I**) Young’s modulus of the hyphal pellicles before and after yeast integration. Data are presented as means ± SD. Statistical significance was determined by two-tailed Student’s *t* test. n.s., not significant; ***P < 0.001. (A) Illustration credit: L. Chen.

Stable integration of the yeast onto the fungal surface was achieved by leveraging the chitin-rich composition of the *C. militaris* cell wall. Chitin is a linear polysaccharide composed of *N*-acetylglucosamine units and can interact strongly with diverse protein- or polysaccharide-binding domains (*53*). We exploited this characteristic by introducing a chitin-binding domain (CBD), named ChBD2 from *Thermococcus kodakarensis*, into the yeast genome via fusion with a glycosylphosphatidylinositol (GPI) anchor under the control of the strong, constitutive *pTDH3* promoter. This modification enabled extracellular secretion and surface presentation of CBD proteins on yeast cells, resulting in stable yeast-hyphae attachment that persisted under prolonged aqueous immersion with negligible detachment (Fig. 3A and fig. S23). Light microscopy further confirmed that CBD-displaying yeast cells were evenly distributed across the hyphal surface, whereas high-resolution SEM revealed uniform layers of yeast intimately associated with the hyphae (Fig. 3B). In contrast, yeast cells lacking CBDs showed uneven coverage and were easily dislodged, highlighting the crucial role of CBDs in maintaining firm yeast-hyphae interactions. Unless otherwise specified, all subsequent experiments used CBD-displaying yeast. To further challenge the robustness of this interaction, we then subjected the composites to 6 hours of ultrasonic treatment and monitored cell detachment via optical density at 600 nm (OD_600_) measurements. CBD-expressing yeast retained ∼75% binding efficiency, whereas that of non-CBD controls decreased to 18% (Fig. 3C), highlighting the essential role of CBD in maintaining coculture integrity.

To assess the versatility of our coculture platform for functional coloration, we engineered four distinct *S. cerevisiae* strains, each designed to produce a specific color pigment (*54*). Specifically, we constructed strains that expressed α-galactosidase (Yeast [Mel1]), β-lactamase (Yeast [BLA]), β-carotene biosynthesis genes (Yeast [Carotene]), and violacein biosynthesis genes (Yeast [Violacein]; Fig. 3, D and E). These strains produced distinguishable colors either through enzymatic conversion of externally added chromogenic substrates (Mel1 and BLA) or via de novo metabolic pathways (carotene and violacein). The resulting colors were pale blue (Mel1), red (BLA), orange (carotene), and deep purple (violacein), confirming the pigment identity and stability under culture conditions (Fig. 3F and fig. S24). By mixing selected pigment-producing yeast strains at defined ratios, we achieved composite intermediate hues spanning red to purple, underscoring the nuanced color control that is often critical for fabric applications. Pigment production was first validated in pure yeast cultures to confirm stable color outputs. Subsequently, the engineered cells were cocultivated with glycerol-treated hyphal pellicles during the predrying or molding phase. The resulting yeast-mycelium composites maintained the flexibility conferred by glycerol (Fig. 3G) while enabling biologically mediated coloration of the fungal matrix, effectively eliminating the need for postproduction dyeing processes known for high water and chemical consumption.

To demonstrate the practical feasibility of our self-dyeing fungal composite, we fabricated a blue-colored textile prototype by coculturing *C. militaris* with α-galactosidase–expressing *S. cerevisiae* and introducing X-α-Gal as the chromogenic substrate. The resulting living fabric exhibited bright and uniform blue pigmentation across the surface, retained its flexibility, and maintained physical integrity under routine folding and handling (Fig. 3H). Given that pigment biosynthesis occurs before integration and that embedded yeast cells become metabolically limited within the mature mycelial matrix, the functional durability of the system depends primarily on the stability of the established coloration rather than continued pigment production. We therefore evaluated temporal color stability following pigment formation. Quantitative image analysis at days 0, 7, and 14 revealed a gradual decrease in overall color intensity, whereas color saturation remained largely unchanged (fig. S25). These results indicate that the pigmentation stabilizes as an embedded material property over time rather than undergoing continuous metabolic amplification or rapid degradation.

Given that industrial-scale textile processing involves substantial mechanical stress, we further assessed whether embedded yeast cells remained stably bound within the material. Tensile testing showed that yeast-laden pellicles exhibited a Young’s modulus of 0.18 ± 0.02 GPa and fracture strain of 42.1 ± 4.7%, closely matching control fungal pellicles (0.20 ± 0.01 GPa and 40.5 ± 3.5%; Fig. 3I and fig. S26), indicating that functional integration does not compromise mechanical performance. Together, these results highlight the synergy between fungal biotechnology and microbial engineering for creating sustainable and high-performing fabric materials.

### Postgrowth functionalization via aerial-hyphae interfaces

**Fig. 4.**
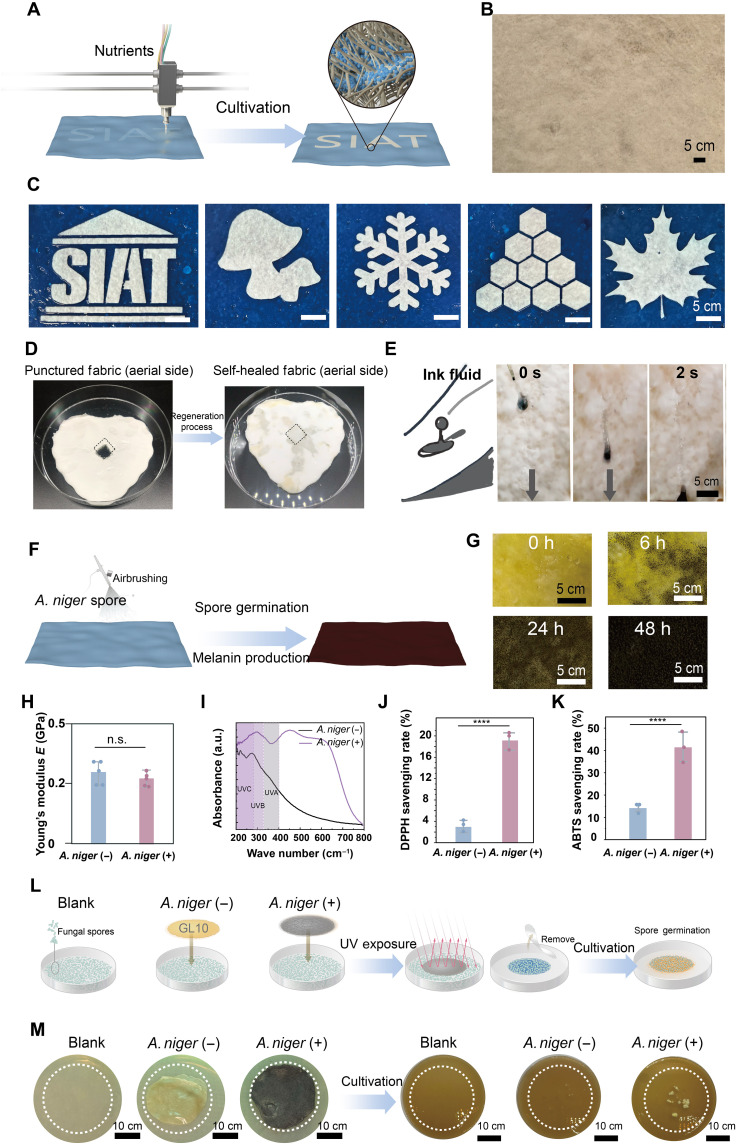
Protective surface functionalization via nutrient-induced aerial hyphae (*C. militaris* and *A. niger*) growth. (**A**) Schematic illustration depicting the hyphal material’s capability to form aerial hyphae upon the application of nutrient solution droplets. (**B**) Top view of a GL10 fungal pellicle fully covered with aerial hyphae after uniform nutrient solution application. (**C**) Optical images showing the growth of aerial hyphae in diverse patterns, including a logo representing Shenzhen Institute of Advanced Technology (SIAT), as well as complex shapes, such as mushrooms, snowflakes, honeycombs, and leaves, demonstrating the programmable nature of the material. (**D**) Repair of the defect achieved by filling the gap with freshly prepared mycelial pellets, followed by incubation under humid conditions with nutrient supplementation. Nutrients provided at the repair site promoted active regrowth of aerial hyphae, seamlessly bridging the gap. After drying, the regenerated area displayed continuous hyphal coverage indistinguishable from the original surrounding material. (**E**) Time-lapse images of wastewater droplets on the aerial hyphae-coated surface, showing hydrophobic behavior and complete droplet removal without residue. (**F**) Schematic diagram of the airbrushing technique used to apply *A. niger* spores mixed with nutrient solution onto the material surface, enabling controlled and uniform spore deposition and subsequent hyphal colonization. (**G**) Optical image showing the uniform hyphal layer formed after airbrushing with *A. niger* spores. h, hours. (**H**) Comparison of Young’s modulus for the hyphal material before and after aerial hyphae growth. (**I**) UV-vis spectra of GL10 and hyphae-coated GL10 with strong UV absorption. a.u., arbitrary units. (**J** and **K**) Antioxidant activity measured by DPPH and ABTS assays. (**L** and **M**) UV-shielding assay demonstrating fungal spore protection under hyphae-coated GL10. Statistical significance was determined by two-tailed Student’s *t* test. n.s., not significant; ****P < 0.0001. (A and L) Illustration credit: L. Chen.

Beyond serving as self-dyeing substrates, fungal viability enabled the induction of aerial hyphae as a downstream functional layer*.* To illustrate this capacity for sustained growth, we stimulated aerial hypha formation by depositing nutrient solution droplets in predefined patterns. Because the embedded mycelium remains viable, these localized nutrients trigger *C. militaris* hyphae to germinate and extend outward, allowing the creation of controlled patterns on specific areas of the pellicle (Fig. 4, A to C). Notably, we successfully induced the Shenzhen Institute of Advanced Technology (SIAT) logo entirely in aerial hyphae by manually depositing nutrient droplets in the shape of the target pattern. Following pattern formation, samples were dried at 60°C to suppress further biological activity. Under high-humidity storage [∼99% relative humidity (RH), ∼19°C], patterned features remained stable for 14 days without detectable boundary blurring (fig. S27).

To restrict undesired lateral expansion of hyphal growth and preserve pattern fidelity during the active growth stage, the surrounding area was bordered with adhesive polypropylene tape serving as a physical barrier. Without such confinement, aerial hyphal growth remains regulatable but forms softer, gradient-like boundaries (fig. S28). These results demonstrate that the living textile retains biological responsiveness postfabrication and can be programmed into user-defined patterns through minimal, low-tech intervention. This patterning capability is complemented by a robust regenerative property: Damaged fungal textiles, such as those with mechanical defects, could be efficiently repaired by placing fresh mycelial pellets at the injury site. Subsequent nutrient-induced aerial hyphae regrowth seamlessly restored both structural continuity and self-cleaning functionalities without the need for external adhesives or coatings (Fig. 4D). Even after up to 10 repeated repair cycles, the regenerated aerial hyphae maintained high hydrophobicity, with WCAs remaining ∼145°, indicating durable restoration of surface functionality. Mechanical testing further showed that the repaired materials retained ∼89% of their original Young’s modulus after 10 cycles (fig. S29). This biologically driven self-repair approach provides clear advantages over traditional synthetic materials, which typically necessitate reapplication of coatings or complete replacement after damage.

Self-cleaning surfaces, especially superhydrophobic coatings, have been widely applied in textiles, architecture, and consumer products due to their ability to repel contaminants and reduce maintenance needs. To construct self-cleaning surfaces on pellicles, we used the self-growing feature of aerial fungal hyphae. The aerial structures grown on pellicles displayed a WCA of ∼145°, indicating strong liquid repellency (figs. S30 and S31). The high contact angle was largely retained after repeated wetting-drying cycles, prolonged soaking (up to 24 hours), and compressive deformation, with only a moderate decrease observed under abrasion (fig. S32). When wastewater droplets were applied to these hyphae-laden regions, they remained spherical and rolled off swiftly, leaving negligible residue. Even when the pellicle was tilted, the droplets slid away without wetting the surface (Fig. 4E). This intrinsic self-cleaning property negates the need for additional chemical coatings, thus simplifying maintenance for self-cleaning fabrics and other protective applications. Notably, our biologically derived strategy simply leverages the natural hydrophobicity and microscale architecture of aerial fungal hyphae to achieve effective self-cleaning without any synthetic coating or patterning (*55*). In contrast, many conventional self-cleaning technologies heavily rely on fluorinated compounds, complex fabrication processes (e.g., photolithography and electrospinning), or surface energy modifiers that are costly, environmentally harmful, or mechanically unstable under real-world conditions (fig. S33) (*56*, *57*).

To further explore the modular potential of our fungal material platform, we investigated whether functional layers could be introduced postfabrication using other fungi with distinct biological traits. We used *A. niger*, a filamentous fungus known for its high melanin content, as an alternative surface modifier. In this complementary procedure, *A. niger* spores were uniformly sprayed onto the pellicle with a nutrient solution (Fig. 4, F and G). The spores germinated across the material’s surface, adding a hyphal layer that augmented its protective traits. Notably, mechanical testing before and after hyphal growth revealed only negligible changes in Young’s modulus and tensile strength (Fig. 4H and fig. S34), indicating that the added biomass did not compromise the pellicle’s structural integrity.

One key motivation for introducing *A. niger* was to endow the pellicle with enhanced UV-protective properties. UV shielding is critically important for textiles used in outdoor environments, where prolonged exposure can degrade fibers, cause discoloration, and accelerate material fatigue. Traditional UV-protective fabrics often rely on embedded nanoparticles or chemical additives such as TiO_2_, ZnO, or benzotriazoles, which may involve multistep processing, environmental concerns, or limited stability under prolonged use (*55*, *58*). In contrast, our approach achieves UV attenuation through biologically grown structures. The abundant melanin pigments present in *A. niger* hyphae are known to effectively attenuate UV radiation, thereby conferring an additional layer of protection (*47*). UV-visible (UV-vis) spectroscopy of the coated material (Fig. 4I) showed markedly increased absorption in the UV range, thus reducing UV transmittance. Furthermore, antioxidant assays [2,2-diphenyl-1-picrylhydrazyl (DPPH) and 2,2′-azino-bis(3-ethylbenzothiazoline-6-sulfonic acid) (ABTS)] revealed that these hyphae-coated pellicles displayed a strong radical scavenging efficiency (Fig. 4, J and K). As standardized indicators of intrinsic antioxidant capacity, these measurements suggest that the melanin-rich coating contributes to enhanced oxidative stability, offering a natural alternative to synthetic antioxidants such as butylated hydroxytoluene or hindered phenols, which may leach or pose toxicity risks (*59*).

To confirm this protective effect under more realistic conditions, we covered potato dextrose agar (PDA) plates containing fungal spores with the hyphae-coated hyphal pellicle and exposed them to 30-W, 254-nm UV light for 3 hours. Spores shielded by the hyphae exhibited near-normal germination, whereas those left unprotected had markedly diminished germination rates (Fig. 4, L and M). This outcome underscores the robust UV-shielding capacity of aerial hyphae in scenarios requiring biological protection. Overall, these adaptive, living modifications yield hyphal fabrics that are self-cleaning, UV-protective, and antioxidant, suggesting exciting possibilities in outdoor gear, biomedical devices, or specialized packaging.

### Toward circular fashion with living fungal textiles

**Fig. 5.**
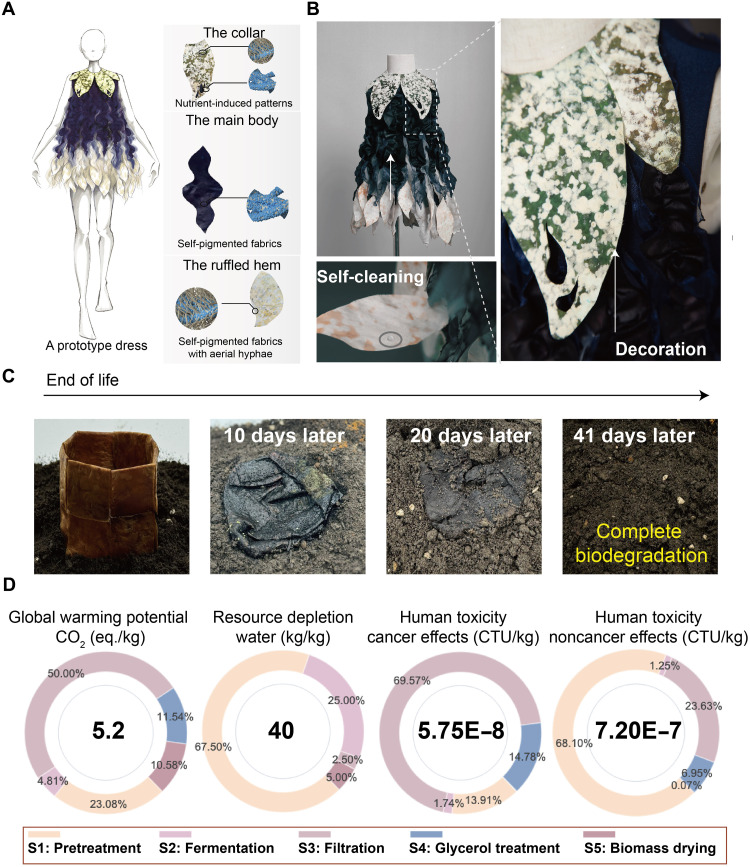
Sustainable living textile prototype with integrated biological functionalities. (**A** and **B**) Prototype garment fabricated from self-pigmented fungal textiles. Engineered *S. cerevisiae* provides in situ coloration, and aerial hyphae confer ultraviolet resistance and self-cleaning properties. (**C**) Soil burial tests demonstrate material near-complete degradation over 41 days. (**D**) LCA results identifying the primary environmental impact categories and key contributors. CTU, comparative toxic unit. (A) Illustration credit: L. Chen.

The viability-preserving strategy enables the fabrication of functional prototypes that demonstrate the potential of fungal textiles for sustainable fashion. Using glycerol-treated pellicles, we produced a garment prototype that incorporated pigment-producing yeast for coloration and aerial hyphae for surface functionality. The living composite maintained flexibility and structural integrity during handling, illustrating the feasibility of translating laboratory-scale materials into wearable formats. The production process began with the formation of GL10 fungal films, prepared by shaping and drying hyphal pellets cultured under optimized conditions. Engineered *S. cerevisiae* strains were cocultured with the pellicle before drying to enable self-pigmentation, while postfabrication nutrient stimulation induced aerial hyphae growth on selected textile modules. The dress was subsequently assembled by sewing distinct fabric modules, each tailored with specialized characteristics. Specifically, the collar consists of approximately four leaf-shaped fungal fabrics, which exhibit irregular, nutrient-induced speckled patterns. The main body of the dress comprises ∼20 modules produced from fungal fabrics cocultured with engineered *S. cerevisiae*, resulting in consistent, self-pigmented blue shades. The ruffled hem consists entirely of petal-shaped fabrics densely covered by aerial hyphae, imparting pronounced self-cleaning properties and textured aesthetics (Fig. 5, A and B). Collectively, these integrated fabric modules demonstrate the versatility and customizable nature of fungal-based living textiles for wearable applications.

We next explored the biodegradability of the material as a key factor in long-term sustainability. As a preliminary test, a small box constructed from GL10 pellicle showed near-complete degradation within 41 days when buried in the soil (Fig. 5C), indicating substantially reduced environmental persistence compared to conventional synthetic polymers. To quantify the broader environmental implications of producing these fungi-derived textiles, we performed LCA in alignment with ISO 14040 and ISO 14044 standards. The analysis encompassed pretreatment (S1), fermentation (S2), filtration (S3), glycerol treatment (S4), and biomass-drying steps (S5; Fig. 5D and fig. S35), with 1 kg of dried fungal biomass set as the functional unit. Impact metrics included the global warming potential (GWP), acidification potential (AP), eutrophication potential (EP), and water use (WU). Notably, the pretreatment stage (S1) accounted for 67.1% of the water consumption, 47.9% of AP, and 45.3% of EP, reflecting substantial nutrient and water requirements. Filtration (S3) was the main driver of the GWP (48.4%) and human toxicity cancer effects (69.5%), primarily due to waste medium disposal. The glycerol treatment step (S4) accounted for 64.4% of the ozone depletion potential (ODP) and 70.3% of land use (LU) impacts, underscoring the resource demands tied to glycerol production (figs. S36 and S37). An accompanying cost analysis revealed that raw materials, particularly nutrient media, made up >95% of production expenses, with glycerol treatment markedly increasing overall costs (table S3).

Collectively, these findings establish a circular framework for living fungal textiles: growth-enabled fabrication, postgrowth functionalization, and end-of-life reintegration into the biosphere. Beyond a single demonstration, the approach defines intervention points for broader deployment, including optimization of pellet morphology for scale-up, substitution of plasticizers, and modular incorporation of diverse microbial partners. This positions fungal textiles as a programmable and sustainable material platform for next-generation circular fashion.

## DISCUSSION

This study establishes a fabrication paradigm that preserves cellular viability to create freestanding fungal textiles, thereby integrating structural integrity with programmable biological functions. By leveraging the innate growth characteristics of *C. militaris* and applying synthetic biology tools, we have engineered macroscale, flexible living scaffolds that maintain metabolic viability and support continued development and postassembly functionalization. This sustained viability enables modular functionalization, as evidenced by two distinct applications: in situ pigmentation through cocultured, engineered *S. cerevisiae*, and surface remodeling via nutrient-induced aerial hyphae formation, which confers emergent properties such as self-renewal and UV protection. These observations highlight a defining feature of filamentous fungi as an ELM chassis. In this framework, the mature mycelial network provides mechanical stability, while retained biological activity enables adaptive and customizable functionalities. Consistent with this view, comparative studies indicate that the capacity for aerial hypha formation is broadly conserved among filamentous fungi, although the underlying regulatory pathways and resulting morphologies are species and strain dependent (*60*), thereby positioning fungal systems as an especially attractive platform for ELMs.

From an application perspective, the intrinsic coupling between biodegradability and biological activity defines distinct deployment scenarios for fungal living textiles. Rather than targeting long-lifetime wearable garments, these materials are more naturally suited to short-term or single-use textile-like systems where environmental sustainability is prioritized. Representative use contexts include biodegradable packaging, temporary exhibition or artistic textiles, and architectural display installations, settings in which conventional materials often persist environmentally despite brief functional lifetimes. In such scenarios, the capacity to deliver programmed functionality during use while enabling controlled end-of-life degradation constitutes a strategic advantage rather than a limitation.

At the same time, the very properties that enable this sustainable, transient use also reveal important bottlenecks that must be addressed before broader adoption in everyday textiles. These include mechanical durability, environmental sensitivity to humidity and temperature fluctuations, and performance variability associated with dynamic biological states. In the context of living textiles, additional metrics such as washability, abrasion resistance, breathability, and wearer comfort have not yet been systematically evaluated and will require further co-optimization of biological and material parameters for practical translation. For example, washability and abrasion resistance could be improved by integrating fungal pellicles into composite laminates with biodegradable reinforcing fibers. Likewise, breathability and wearer comfort could be tuned by controlling pellicle thickness and porosity, or combining the living layer with open-weave textile substrates to maintain air and moisture transport.

Looking forward, the integration of synthetic biology into living textiles opens several compelling research avenues. In the present system, engineered *S. cerevisiae* functions as a spatially confined and nutritionally restricted module within the mycelial matrix, inherently limiting proliferation and environmental persistence (*11*). While this modular coculture strategy enables flexible postgrowth functionalization, future efforts could move toward more direct engineering of fungal mycelium itself. Endogenous genetic modification of the mycelial chassis would allow intrinsic production of functional molecules, such as pigments, antimicrobial compounds, or enzymes, thereby simplifying system architecture, improving functional integration, and reducing reliance on auxiliary microbial partners. The development of CRISPR-based tools and synthetic gene circuits would further allow precise spatiotemporal control over these traits, facilitating material responses to environmental signals or damage.

In conclusion, this work establishes a scalable framework for constructing fungal ELMs that integrate growth-driven fabrication, modular functionalization, and programmed biodegradability. By bridging structural formation with sustained metabolic activity, we introduce a class of biofabricated systems that are programmable, environmentally responsive, and intrinsically sustainable. Beyond textile analogs, this paradigm provides a foundation for the broader development of adaptive, living material platforms across biomedical, environmental, and architectural domains.

## MATERIALS AND METHODS

### Materials and reagents

All chemicals and reagents were purchased from Sigma-Aldrich, unless otherwise noted. Culture-grade glycerol, yeast extract, peptone, PDA, and other standard medium components were used without further purification. Molecular biology reagents, including restriction enzymes, T4 ligase, polymerases, and DNA purification kits, were obtained from New England Biolabs (NEB) or Takara. Deionized water (18.2 MΩ·cm) was used for all buffer and medium preparations. All solutions were sterilized by autoclaving or filtered with 0.22-μm filtration.

### *C. militaris* cultivation and pellet optimization

*C. militaris* was obtained from the China Center of Industrial Culture Collection and maintained on PDA slants at 4°C. For submerged culture, fungal spores were inoculated into 50 ml of potato dextrose medium without agar in 250-ml flasks and agitated at 150 rpm. To determine optimal culture conditions for mycelial pellet formation, three key parameters were varied systematically: initial spore concentration (10^4^ to 10^8^ spores/ml), pH (*2*–*10*), and incubation temperature (16° to 32°C). All cultures were harvested after 72 hours, a cultivation window commonly reported to yield morphologically mature and uniform fibrous pellets in filamentous fungi, suitable for downstream material assembly and characterization (*47*, *61*). Pellets were filtered and washed with deionized water before further processing.

### Yeast strain construction and genetic engineering

#### Yeast strain and culture conditions

All experiments used the *S. cerevisiae* BY4741 strain (MATa his3Δ1 leu2Δ0 met17Δ0 ura3Δ0 sed1Δ) as the primary host. Cultures were propagated in yeast extract–peptone–dextrose (YPD) medium, formulated with 1.5% (w/v) yeast extract (Angel Yeast), 2.0% (w/v) peptone (Angel Yeast), and 2% (w/v) glucose (Sangon).

#### Plasmid construction for pigment synthesis

Pigment-producing plasmids were built to express β-carotene synthesis genes (*crtYB*, *crtI*, *crtE*, and *tHMG1*) or violacein synthesis genes (*vioA*, *vioB*, *vioC*, *vioD*, and *vioE*), as well as enzymes for reporter assays (Mel1 and BLA). These genes were placed under constitutive promoters (*pTDH3*, *pTEF1*, *pCCW12*, and *pTEF2*) for robust expression in *S. cerevisiae*. Construction used a Golden Gate approach (Bsa I based), assembling each gene cassette into the final plasmid. Four pigment-producing *S. cerevisiae* strains were constructed to introduce distinct color profiles directly onto the fungal pellicle: Yeast [Mel1] encodes α-galactosidase under *pTDH3* promoter, producing a pale blue hue upon substrate conversion. Yeast [BLA] encodes β-lactamase (TEM-1), generating a red chromogenic signal with appropriate substrates. Yeast [Carotene] contains carotenoid synthesis genes (*crtE*, *crtYB*, *crtI*, and *tHMG1*), yielding an orange pigment. Yeast [Violacein] expresses VioABCDE from *Chromobacterium violaceum*, imparting a deep purple coloration.

#### Construction of GPI-anchored protein plasmids

To facilitate cell surface anchoring and chitin binding, a CBD from *T. kodakarensis* was appended to the C terminus of selected GPI-anchored proteins. These fusion constructs were created through Golden Gate cloning. The engineered yeast strains and their corresponding pigment or anchoring functions are summarized in table S4, and full plasmid sequences are documented in table S5*.*

### Coculture of *S. cerevisiae* and *C. militaris*

Each yeast strain was grown overnight (OD_600_ of ∼0.8) in YPD medium at 30°C with shaking at 200 rpm. Cells were harvested by centrifugation (5000*g*, 5 min), washed twice in PBS, and resuspended to ∼1 × 10^7^ cells/ml. *C. militaris* was first cultured in a potato water medium at 25°C and 120 rpm for 48 hours to allow mycelial growth.

After 48 hours, an excess amount of genetically engineered *S. cerevisiae* was added to the culture. The coculture was incubated for an additional 12 hours under the same shaking conditions. Following incubation, the mixture was subjected to 6-hour sonication using an ultrasonic cleaning bath (Fuyang F-060SD, 15-liter capacity) operated at a working frequency of 40 kHz and a nominal ultrasonic power of 240 W. Samples were sealed in containers and immersed in the ultrasonic bath under continuous operation (continuous duty cycle). The bath temperature was maintained at room temperature without activating the heating function to minimize thermal effects during sonication. The total sample volume subjected to sonication was 45 ml. The culture was then filtered through two layers of sterile gauze to remove excess medium and unattached yeast cells, leaving the bound yeast-hyphae composite for further use.

### Aerial hyphae growth induction

Two methods were used to induce aerial hyphae on glycerol-treated *C. militaris* pellicles (GL10s). First, for patterned growth, a potato water medium was carefully dropped onto defined zones of the pellicle (not subjected to high temperature deactivation) to supply localized nutrients, while adhesive tape was placed around the droplet regions to confine hyphal extension. The samples were incubated in a humid environment (25° ± 2°C, >90% RH) for 72 to 96 hours, after which the tape was removed to reveal precisely formed aerial hyphae patterns. Second, for broad coverage with *A. niger*, the pellicles were heat treated at 120°C to inactivate residual *C. militaris* and then uniformly sprayed with an *A. niger* spore suspension (∼1 × 10^7^ spores/ml). These spore-coated pellicles were placed in a humid chamber (28° ± 2°C) for 48 hours, allowing melanin-rich aerial hyphae to form across the surface, thereby conferring robust UV shielding and a hydrophobic finish. Control pellicles (GL10 without *A. niger*) were subjected to the same incubation conditions but without spores.

### Pellet harvesting and pellicle molding protocol

After 72 hours of submerged culture, fungal pellets were collected via filtration through two layers of gauze and washed with deionized water to remove residual medium. The resultant slurry was drop-cast onto a clean substrate and gently pressed between sterile petri dishes and dried at 45°C for 6 hours to form flat, coherent pellicles.

### Pellet-mediated repair of fungal pellicles

Fungal pellicles were fabricated by assembling freshly harvested mycelial pellets into freestanding films as described above. To assess postfabrication repairability, dried pellicles were mechanically damaged to create cracks or voids of variable size. Localized repair was performed by manually placing freshly harvested mycelial pellets with matched hydration directly into the damaged regions, ensuring contact with the surrounding intact pellicle, without the use of external adhesives or fixation.

The amount of pellets used for repair was scaled with the size of the damaged area, following the same pellet areal density as in the original pellicle fabrication. For example, a pellicle prepared from 500 mg (wet weight) of pellets to form a 25-cm^2^ film was repaired by filling an ∼1-cm^2^ damaged region with ∼20 mg (wet weight) of pellets under identical hydration conditions. For repair assays involving surface-function recovery, repaired regions were further supplied with potato water medium and incubated under humid conditions to induce aerial hyphae regrowth before hydrophobicity and mechanical testing. Repaired pellicles were allowed to dry under ambient conditions before further characterization.

### Glycerol plasticization procedure and parameters

Dried pellicles were soaked in glycerol solutions (0, 10, 30, and 50%) for 24 hours. Samples were then dried under ambient conditions for 24 hours. Pellicles treated with ≥90% glycerol exhibited phase separation when soaked; for these conditions, glycerol was directly blended into the wet pellets before molding and drying.

### Material fabrication and performance evaluation

#### Mechanical testing: Young’s modulus, tensile strength, and ductility

The thickness of the fabrics was measured at three equidistant points along the sample using a digital length gauge. Tensile tests were performed at room temperature using a CMT6103 testing machine (MTS systems Corporation, USA) with a preload force of 0.25 N and a test speed of 2 mm/min. The bulk density of the pellicles was calculated by dividing the sample weight by its bulk volume. Key mechanical parameters, including Young’s modulus, elongation-at-break, and tensile strength were extracted from stress-strain curves.

#### SEM, FTIR, UV-Vis, and surface morphology analysis

##### Scanning electron microscopy

Surface and cross-sectional microstructures of glycerol-treated *C. militaris* hyphal pellicles were characterized using a field-emission scanning electron microscope (Hitachi SU8100, Japan). Before imaging, pellicle samples (∼1 cm by 1 cm) were fixed on aluminum stubs using conductive carbon tape and sputter-coated with a thin layer (∼10 nm) of gold using a magnetron sputter coater (Cressington 108 Auto). Imaging was performed at an accelerating voltage of 15 kV under high-vacuum mode. Both surface and fracture-edge morphologies were imaged at magnifications ranging from ×500 to ×10,000 to visualize hyphal entanglement, glycerol plasticization effects, and crack formation.

##### ATR-FTIR spectroscopy

Chemical functional groups of native and glycerol-treated pellicles were analyzed using an ATR-FTIR spectrometer (Bruker Tensor II, Germany) equipped with a diamond crystal. Dried pellicle samples (∼1 cm^2^) were gently pressed against the crystal surface with a torque-controlled clamp. Spectra were collected in the range of 4000 to 600 cm^−1^ with a resolution of 4 cm^−1^ and 32 scans per sample. Spectral baseline correction and normalization were performed using Bruker OPUS software. Changes in hydroxyl, amide, and C─O vibrational bands were used to assess hydrogen bonding and glycerol interaction with fungal polysaccharides.

##### UV-vis absorption spectroscopy

To evaluate light absorption and UV shielding performance, pellicles (1 cm by 1 cm) were measured using a Shimadzu UV-2600 spectrophotometer. Spectra were recorded in the wavelength range of 200 to 800 nm at 1-nm intervals using an integrating sphere module to accommodate opaque samples. The transmittance of pellicles treated with various glycerol concentrations or coated with aerial hyphae was compared to quantify UV-blocking efficiency.

##### Surface morphology and roughness analysis

Macroscopic surface texture and color uniformity were documented using a digital camera (Nikon D7500) under identical illumination conditions. Images were further processed using ImageJ software to quantify surface roughness parameters such as hyphal network coverage. At least three randomly selected fields of view per sample were analyzed to ensure statistical reproducibility.

#### WCA and solvent resistance test

Wettability of each glycerol-treated *C. militaris* hyphal pellicle was assessed using a 2-μl droplet of deionized water deposited on the sample surface. Measurements were performed with a Drop Shape Analyzer (Kruss JY-82B) under ambient laboratory conditions (25° ± 2°C, 50 ± 5% relative humidity). Before testing, pellicle samples (1 cm by 1 cm, ∼2 mm thick) were gently dusted with compressed air to remove surface debris and equilibrated for at least 30 min in the testing environment. A side-view camera captured the droplet profile immediately (within 2 s) after deposition, and the contact angle (θ) was calculated using the Young-Laplace fitting method provided by the device software. Five independent measurements were conducted on different regions of each sample, and the average value ± SD was reported.

### UV-shielding assay and fungal viability under UV stress

A UV chamber (30 W UV-C lamp, λ = 254 nm) was used for irradiation. Chamber temperature was maintained at 25° ± 2°C. Pellicles were placed inside the chamber at a distance of ∼15 cm from the UV source. PDA plates (90-mm diameter) were evenly spread with ∼1 × 10^5^ spores of *C. militaris* (or an alternative test fungus). The corresponding pellicle circular discs (2 cm) were gently placed over the inoculated PDA on the plate. Plates were then exposed to UV-C light (30 W) for 3 hours in the closed chamber. After irradiation, plates were immediately transferred to a 25°C incubator for 48 hours. Germination rates were recorded.

### Antioxidant capacity: DPPH and ABTS assays

Hyphae-coated fungal pellicles (∼0.5 cm by 0.5 cm, ∼2 mm thick) were carefully cut using sterile scissors or a punch tool. Samples were briefly rinsed with deionized water and gently blotted dry with a lint-free wipe to remove surface debris.

DPPH/ABTS radical scavenging activity was measured with a commercial assay kit (Beijing Solarbio Science & Technology Co. Ltd.). The mixture of samples and DPPH was allowed to stand at room temperature in the dark for 30 min. Then, the absorbance of the resulting solution was measured at 517 nm (DPPH) or 734 nm (ABTS). A lower absorbance indicates higher free radical scavenging activity. The DPPH/ABTS radical scavenging activity of each sample was calculated as the percent inhibition according to the following equations

$$\text{Scavenging rate}\%=\frac{A_{\text{blank}}-\left(A_{\text{sample}}-A_{\text{negative control}}\right)}{A_{\text{blank}}}\times 100\%$$

where *A*_negative control_ represents the absorbance (*A*) of the sample and absolute ethyl alcohol and *A*_blank_ represents the absorbance (*A*) of double distilled water and testing buffer in the kit.

### Final garment assembly

Large-area living fabrics were produced by pouring semi-wet *C. militaris* biomass into PTFE trays measuring 15 cm by 15 cm, with a controlled thickness of ∼2 mm. Glycerol treatment at 10% (GL10) was applied to balance flexibility and mechanical robustness. After drying at 45°C for 6 to 8 hours, each sheet was inspected for uniform thickness, minimal surface cracking, and consistent coloration. In parallel, aerial hypha zones were locally nutrient treated to yield textured, self-cleaning surfaces on the sleeves.

### Biodegradability test

To determine the material’s end-of-life fate under realistic conditions, we examined the soil biodegradation behavior of fabric-based cubic box over a 41-day period. A small cubic box was fabricated from GL10 to further assess biodegradation in a real-world form factor. Loamy soil (pesticide-free) was collected from a local agricultural test field, then air-dried, and sieved (2-mm mesh) to remove large debris. The box was buried horizontally at ∼5-cm depth in plastic trays (30 cm by 20 cm by 15 cm). Each tray contained ∼3 kg of soil, maintained at 50 to 60% water-holding capacity by periodic misting with deionized water. Trays were stored under laboratory ambient conditions (22° to 26°C, RH of ∼50%), simulating typical indoor or covered outdoor scenarios. Every 2 days, the sample was exhumed, gently brushed free of soil and photographed to record morphological changes.

### Life cycle assessment

A LCA was conducted in accordance with ISO 14040 and ISO 14044 guidelines, with the functional unit defined as 1 kg of dried *C. militaris* biomass. The study encompassed five principal process stages: (S1) pretreatment of nutrient medium and water, (S2) submerged cultivation of fungal cells, (S3) biomass filtration and washing, (S4) glycerol treatment of the harvested mycelium, and (S5) drying to obtain the final product. Each stage’s material and energy flows were recorded through direct laboratory measurements and equipment specifications. A cradle-to-gate boundary was set, covering raw material procurement (e.g., yeast extract, peptone, glycerol, and water), energy consumption (e.g., electrical usage for incubation, drying, and pumping), and any residual waste (spent medium and wash water). The eFootprint platform, in conjunction with the Chinese Life Cycle Database (CLCD) and ecoinvent databases, was used to model background processes and derive impact factors. Midpoint indicators evaluated included GWP, AP, EP, ODP, human toxicity (carcinogenic and noncarcinogenic), LU, WU, primary energy demand, and photochemical ozone formation. Mass-based allocation was used where by-products appeared, while emissions and waste streams were assigned on the basis of measured outputs from each stage. Sensitivity analyses were performed by adjusting key inputs (water volume, nutrient medium composition, and glycerol sourcing) to determine how process changes influence the overall environmental profile.

### MD simulations

The AMBER force fields are used in all simulations. Both of these potentials include bonded interactions representing immutable covalent bonds and nonbonded interactions representing long-range van der Waals and Coulombic interactions. In both cases, van der Waals interactions are modeled using a standard 12/6 Lennard-Jones (L-J) pair potential (Eq. 1) with inner and outer cutoff radii of 9 and 10 Å to describe the interactions among solution molecules, and the L-J pair interactions between different atoms were defined using the Lorentz-Berthelot mixing rules (Eq. 2). A shift function is used to ramp the energy and force smoothly to zero between the inner and outer cutoff radii. Periodic boundary conditions are prescribed in all three orthogonal directions. At different stages, the MD simulations are performed either under NPT conditions (constant pressure and temperature) or NVT conditions (constant volume and temperature) as explained below. In LAMMPS (Large-scale Atomic/Molecular Massively Parallel Simulator), temperature is maintained using a Nose-Hoover thermostat with a 100-fs coupling constant, and pressure is maintained with a Nose-Hoover barostat with a 1000-fs coupling constant. Moreover, we used particle-particle particle-mesh (PPPM) algorithm (*62*) on LAMMPS software to compute long-range Coulombic interactions at each timestep.

To obtain stable configurations, we performed annealing process to rectify any initial unrealistic structures, providing a more reasonable equilibrium geometry for the quasistatic tensile simulations. Six annealing periods (about 5 ns) were set for the entire system under the NPT ensemble

$$E_{pot}=\sum_{i>j}4{\text{ε}}_{ij}\left[{\left(\frac{{\text{σ}}_{ij}}{r_{ij}}\right)}^{12}-{\left(\frac{{\text{σ}}_{ij}}{r_{ij}}\right)}^{6}\right]$$ (1)

$$\sigma_{ij}=\frac{1}{2}\left(\sigma_{ii}+\sigma_{jj}\right);\varepsilon_{ij}={\left(\varepsilon_{ii}\varepsilon_{jj}\right)}^{1/2}$$ (2)

In our MD simulations, we conducted quasistatic uniaxial tensile tests on polysaccharide mixtures with varying concentrations of glycerol to investigate the impact of glycerol additives on their mechanical behavior. The uniaxial tension was applied along the *x* axis, with the tensile tests carried out at six different models. The stretching process was performed incrementally. Specifically, tension was achieved by incrementally changing a dimension of the box in the *x* direction at a constant engineering strain rate of 5 × 10^9^ s^−1^, while the dimensions in the *z* and *y* directions remained constant. The strain rate value appears to be significantly higher than experimental values. However, such high strain rates (10^6^ to 10^11^ s^−1^) are commonly used in MD simulations due to computational performance limitations. Subsequently, at each step, the pressure tensor *P_x_* and the cell dimension *L_x_* in the stretching direction were saved. On the basis of the obtained characteristic quantities, relative tensile strain and stress were calculated. After each 0.5-Å stretch, 10,000 steps of NVT ensemble structural relaxation were performed to allow the system to relax. A Nose-Hoover thermostat was used to maintain the system temperature. All simulation results were visualized by the VMD program package.

### Statistical analysis

Unless otherwise stated, quantitative experiments were performed with at least three independent replicates, and data are presented as means ± SD. Statistical tests and sample sizes are specified in the corresponding figure captions.
