# Supplementary material for: A programmable fungal platform for engineered living textiles
Source: Sci Adv. 2026 Jul 24;12(30):eaed6937. doi: 10.1126/sciadv.aed6937 (PMC13398475; doi:10.1126/sciadv.aed6937)
Supplement: Supplementary file 1 [file sciadv.aed6937_sm.pdf]

Science Advances
AAAS

Supplementary Materials for

A programmable fungal platform for engineered living textiles

Ke Li *et al.*

Corresponding author: Bolin An, bl.an@siat.ac.cn; Xinyu Wang, xy.wang3@siat.ac.cn;
Chao Zhong, chao.zhong@siat.ac.cn

Sci. Adv. **12**, eaed6937 (2026)
DOI: 10.1126/sciadv.aed6937

This PDF file includes:

Figs. S1 to S37
Tables S1 to S5

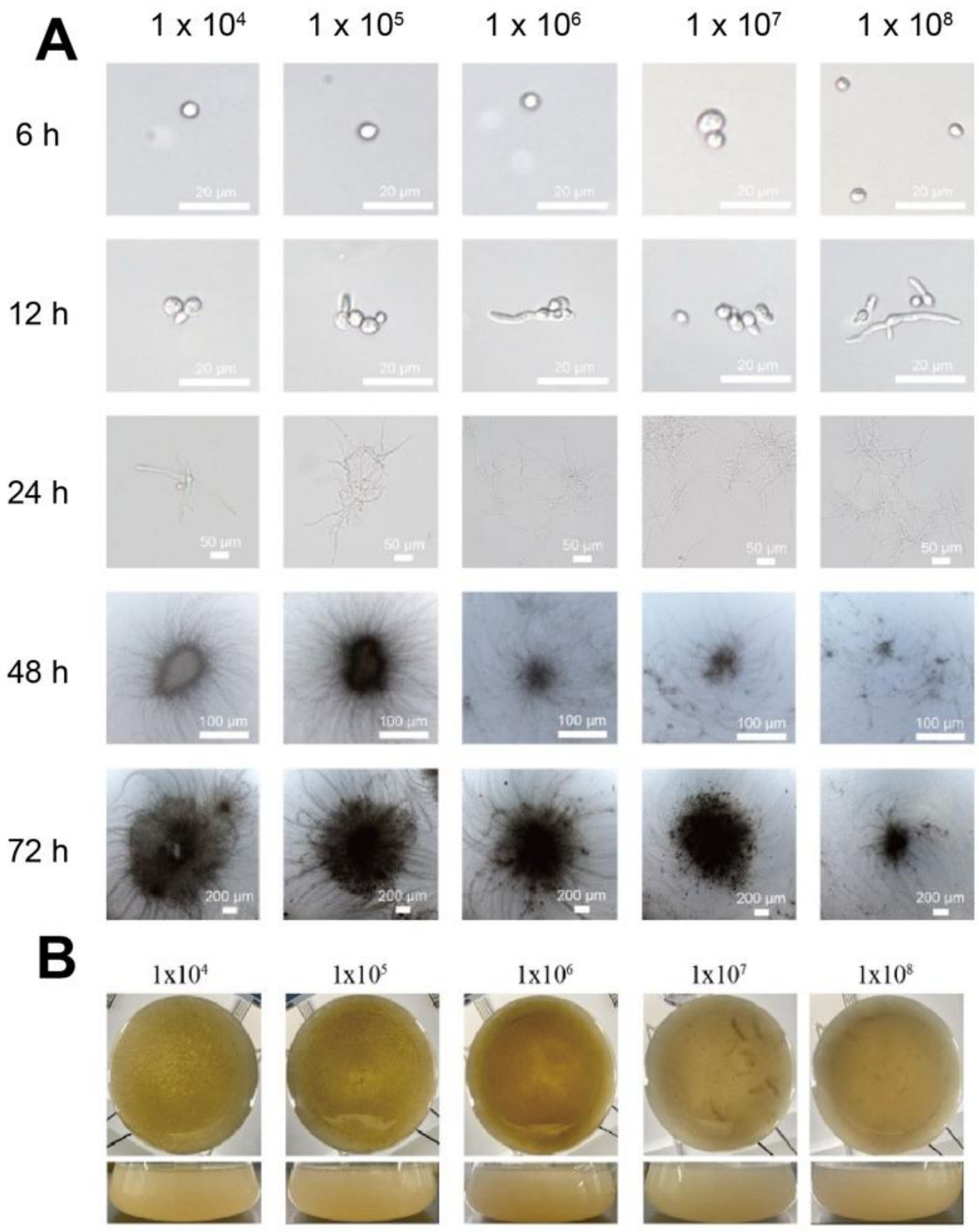

Figure S1 | Effect of initial spore concentration on mycelial pellet formation in submerged *C. militaris* culture. A. Spores of *C. militaris* were inoculated into liquid PDA medium at concentrations ranging from 10^4 to 10^8 spores/mL and cultured under agitation at 150 rpm for 72 hours. **B.** Light microscopy was used to observe the morphology and size distribution of resulting mycelial pellets.

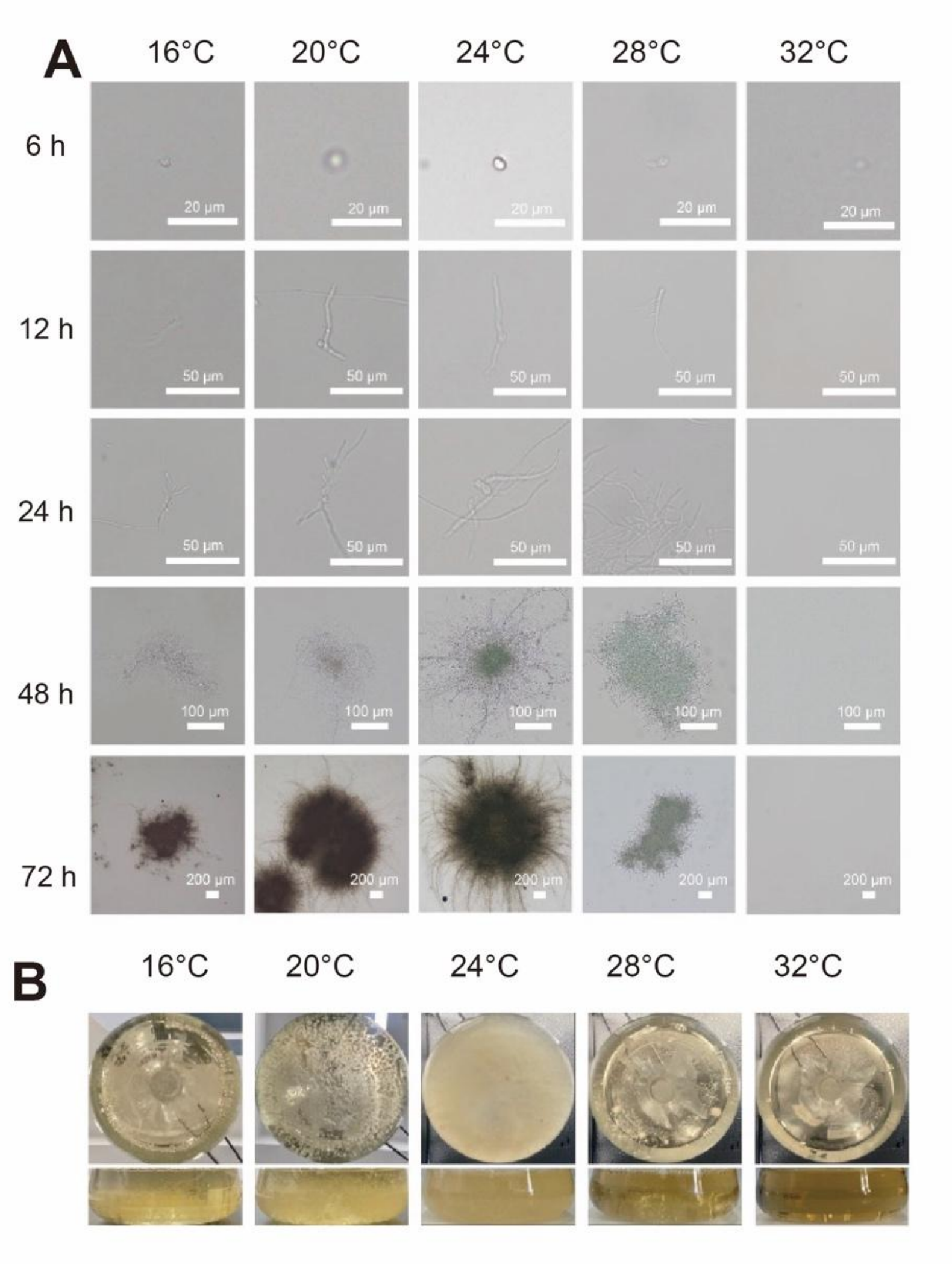

Figure S2 | Effect of incubation temperature on mycelial pellet morphology. A. *C. militaris* spores were cultured in liquid PDA at temperatures ranging from 16 °C to 32 °C for 72 hours. **B.** Pellet morphology was documented under light microscopy. Lower temperatures resulted in slow growth and sparse structures, while higher temperatures produced loosely aggregated, fragmented mycelia.

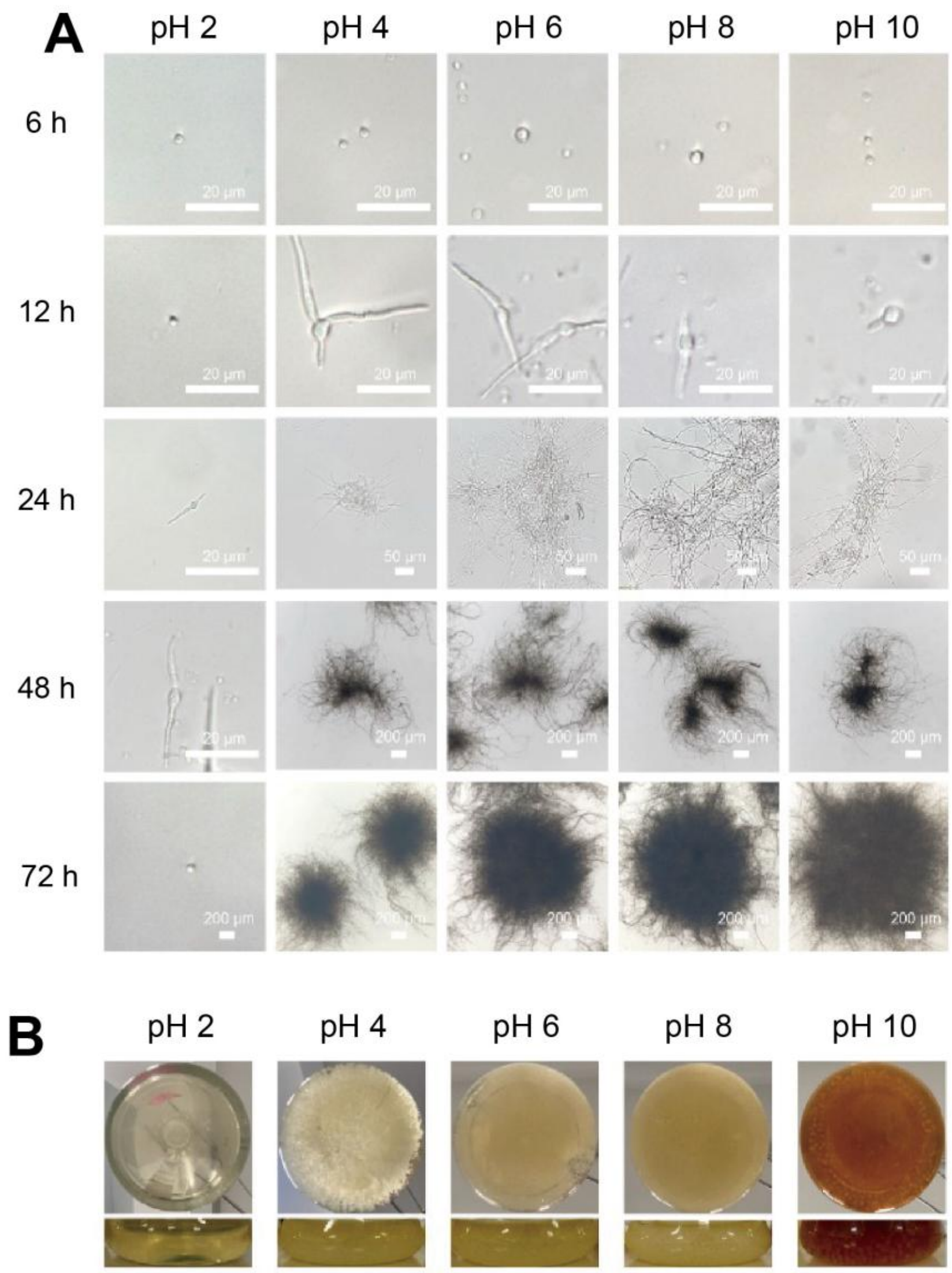

Figure S3 | Influence of culture medium pH on hyphal pellet development. A. Liquid cultures of *C. militaris* were adjusted to pH 2–10 before inoculation and incubated under standard conditions for 72 hours. **B.** Pellets were harvested and examined microscopically. Near-neutral pH (6–8) favored formation of dense, round, and uniform hyphal pellets, while extreme pH values (≤3 or ≥9) led to malformed or dispersed hyphal fragments, compromising pellet integrity.

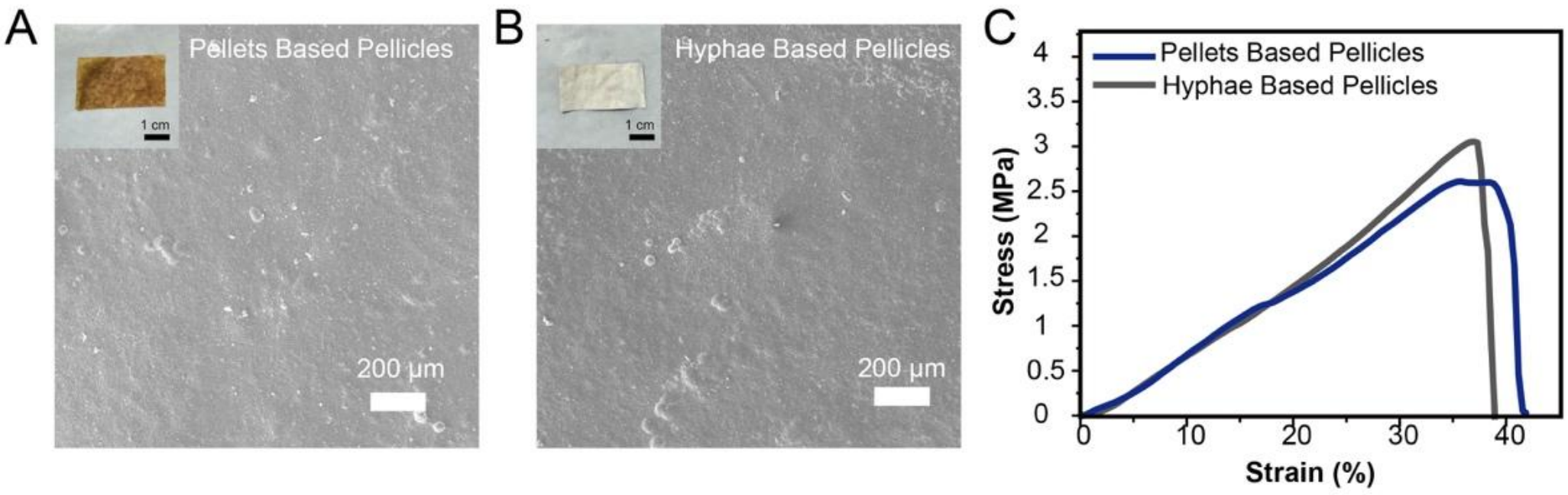

Figure S4 | Structural and mechanical comparison between pellet-based and hyphae-based pellicles. A. Representative SEM image of pellet-based pellicles. Insets show the macroscopic appearance of the corresponding films. **B.** Representative SEM image of hyphae-based pellicles with comparable apparent surface uniformity at the same magnification. Insets show the macroscopic appearance of the aerial hyphae facing surface. **C**. Representative stress-strain curves of pellet-based and hyphae-based pellicles.

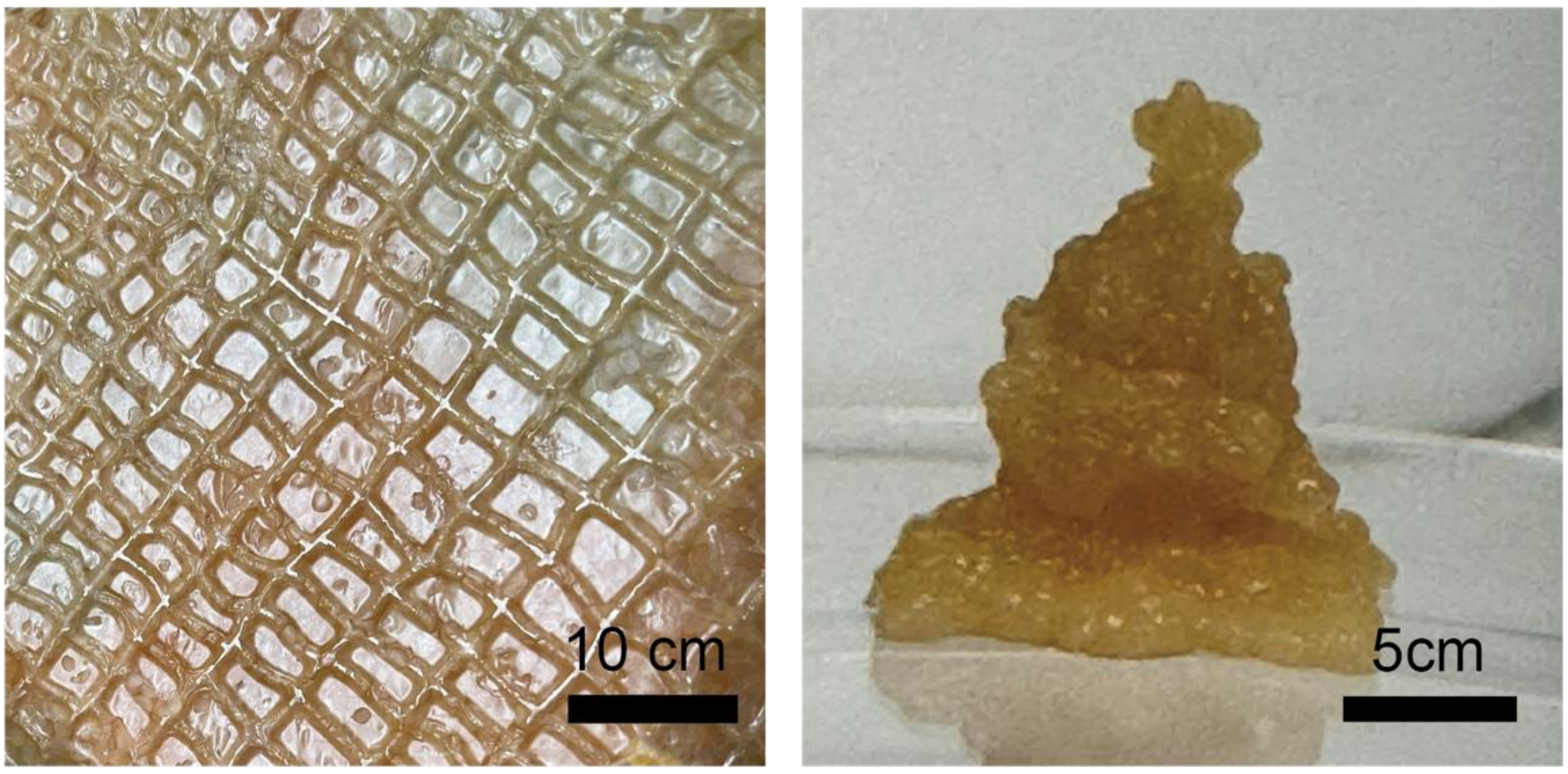

Figure S5 | Demonstration of moldability of pellets into 2D and 3D forms. *C. militaris* pellets were shaped into sheets and complex geometries (folds, tree structure) by casting into molds or direct printing, followed by controlled drying.

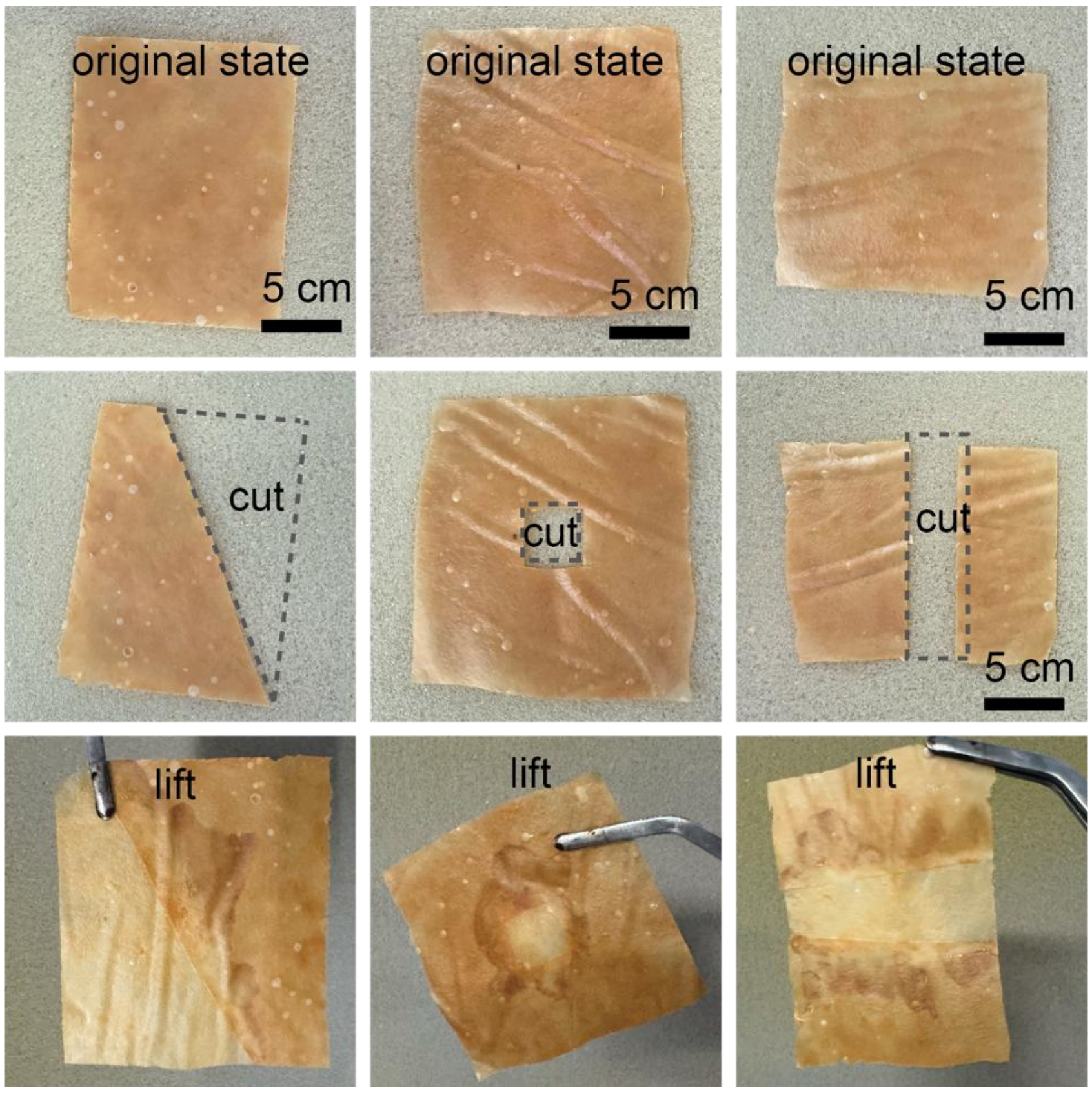

Figure S6 | Repair of mechanically damaged fungal pellicles via localized reassembly using fresh mycelial pellets. Top row: Original pellicles in intact state. Middle row: Deliberately cut samples simulating structural defects (diagonal, central, and vertical incisions). Bottom row: Freshly cultured wet *C. militaris* mycelial pellets were manually deposited at the damaged sites. Upon drying under ambient conditions, the pellets bonded with the surrounding hyphal matrix via interfacial hydrogen bonding, effectively sealing the cuts. This result demonstrates that mycelial pellets function as modular "building blocks" that can be used to repair or extend fungal materials post-fabrication.

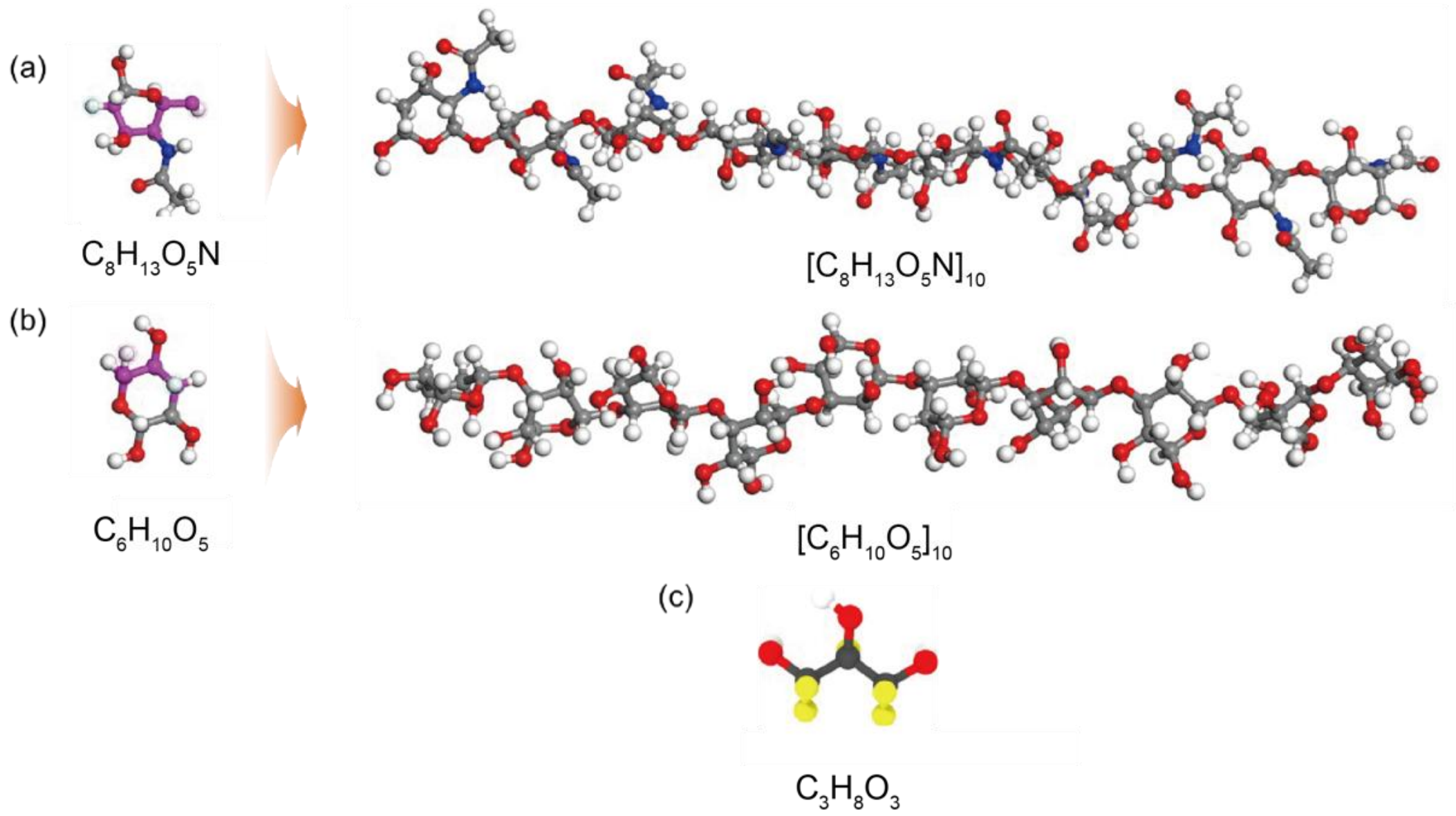

Figure S7 | Initial molecular structures used in MD simulations. Visual representation of modeled $C_8H_{13}O_5N$ (chitin), $C_6H_{10}O_5$ (Glc), and $C_3H_8O_3$ (glycerol) monomers/polymers prior to simulation. These serve as proxies for fungal cell wall components and plasticizer molecules.

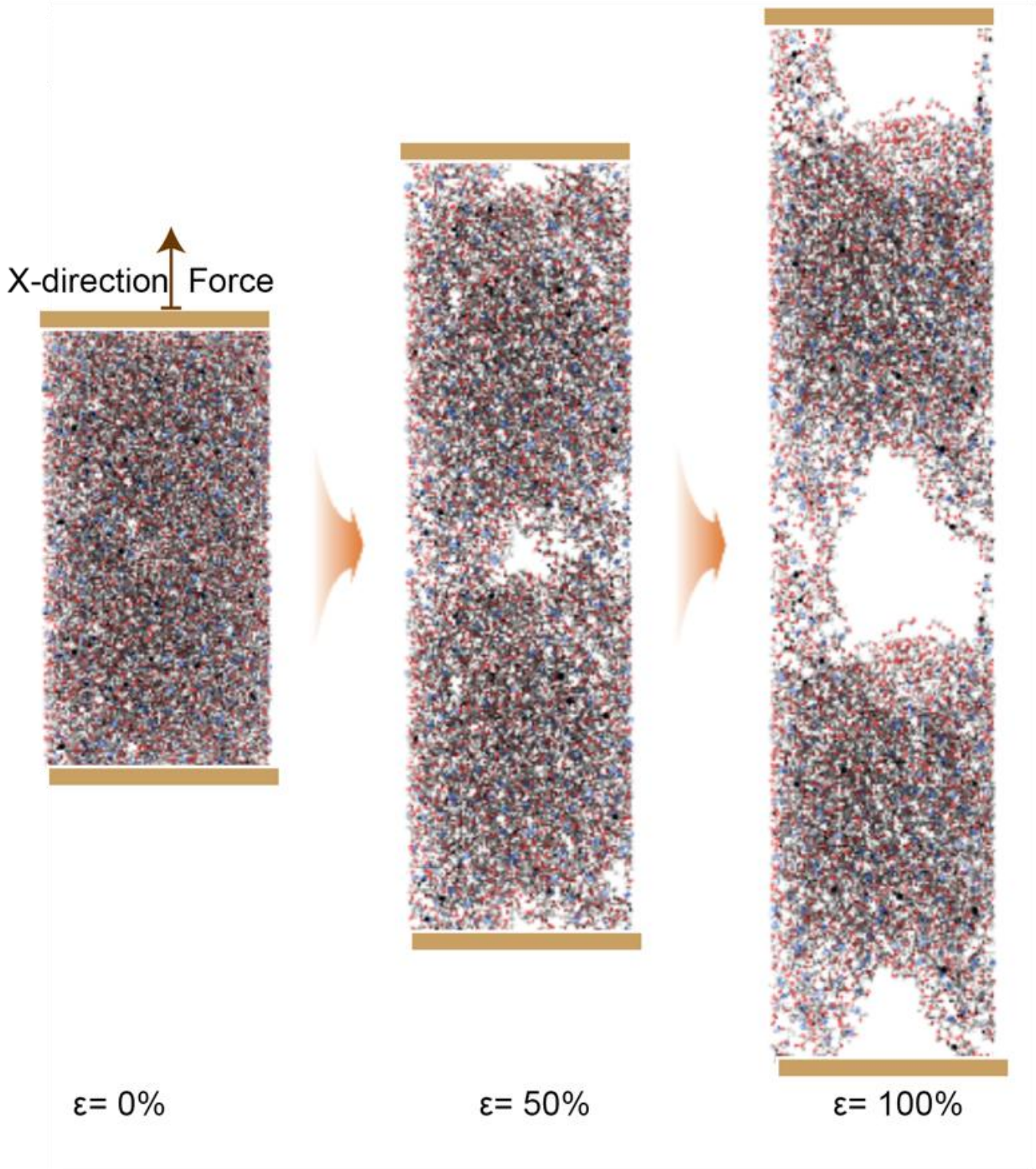

Figure S8 | Snapshots of molecular network evolution under strain. Simulated polysaccharide–glycerol systems were subjected to tensile strain along the x-axis. Atomic configurations are shown at strain = 0, 0.5, and 1.0 to illustrate network deformation. Color codes for each component correspond to the atomic models defined in Figure S7.

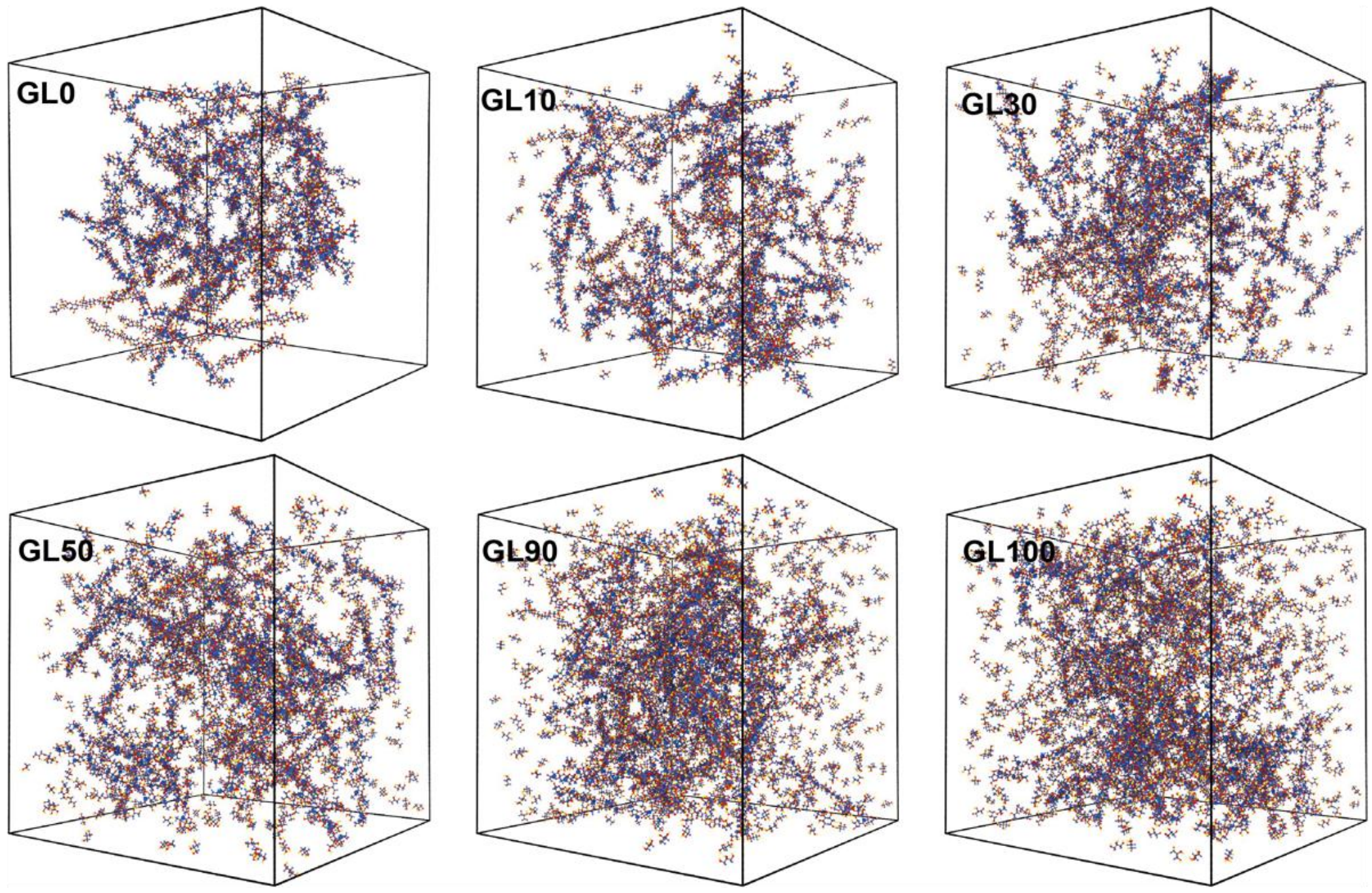

Figure S9 | Simulated glycerol distribution in the polysaccharide matrix. Simulated molecular distribution in the fungal-derived polysaccharide network showing chain packing and glycerol localization. Color codes for each component correspond to the atomic models defined in Figure S7.

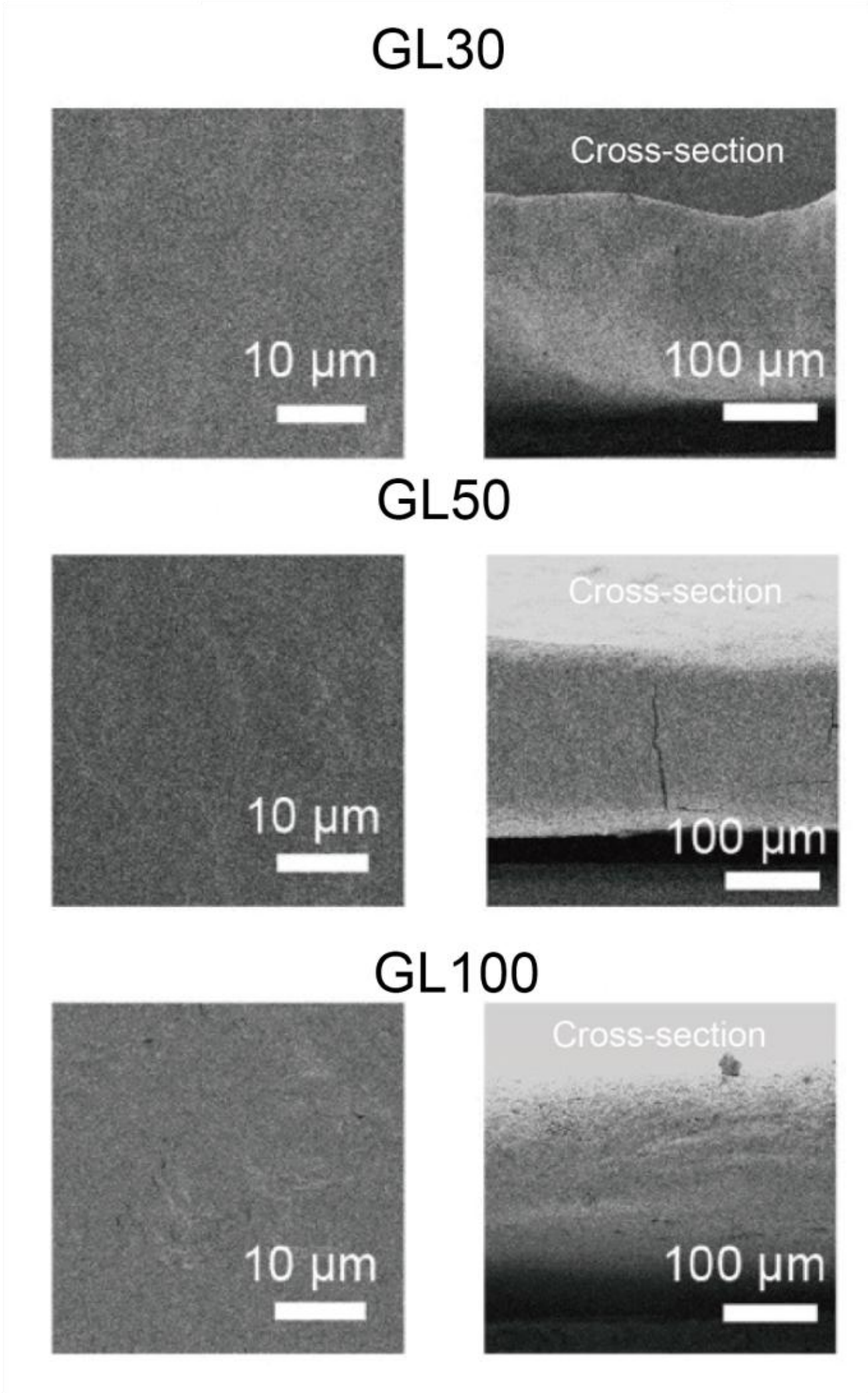

Figure S10 | SEM micrographs demonstrating morphological consistency of fungal pellicles treated with varying glycerol concentrations. SEM images of *C. militaris* hyphal pellicles treated with 30%, 50%, and 100% glycerol reveal similar surface morphologies across these conditions.

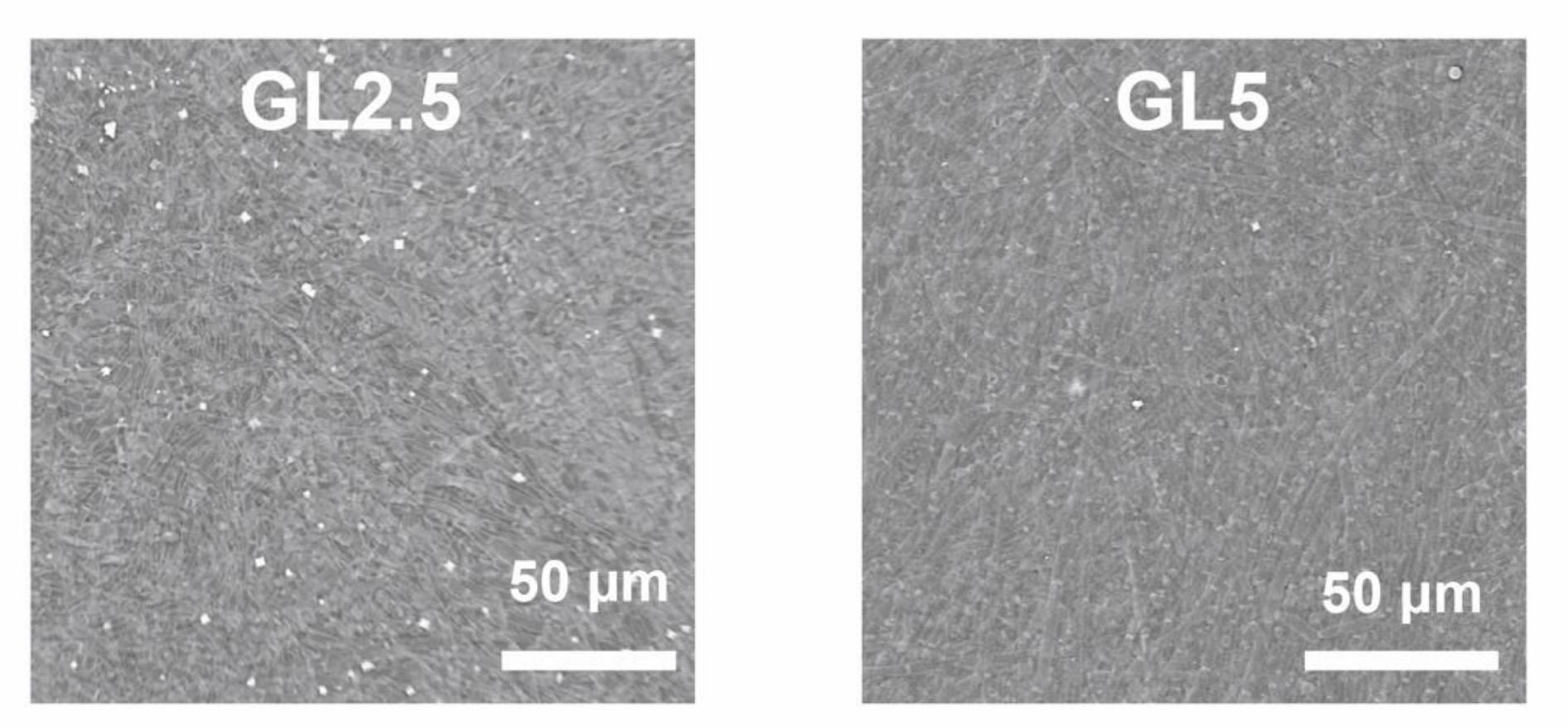

Figure S11 | SEM micrographs demonstrating morphological consistency of fungal pellicles treated with 2.5% and 5% glycerol.

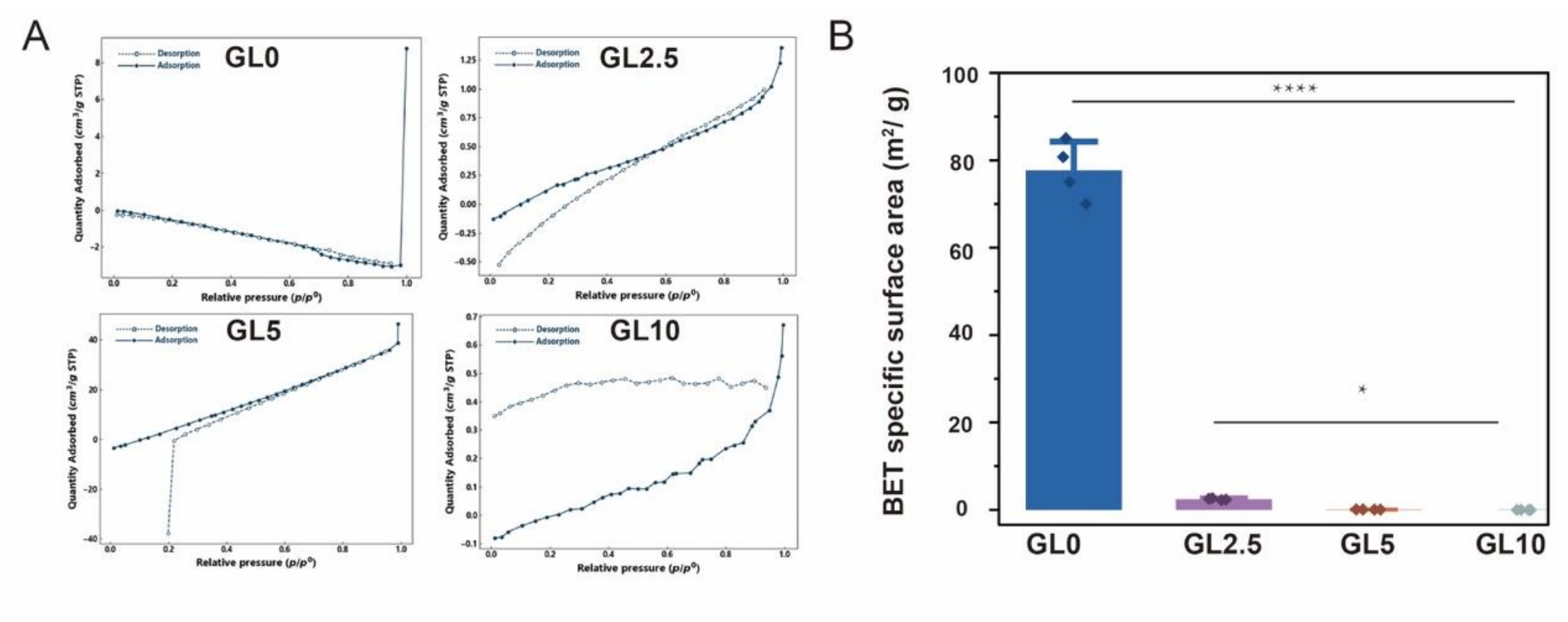

Figure S12 | Effect of glycerol content on pore structure and specific surface area of fungal pellicles. A. Nitrogen adsorption-desorption isotherms of fungal pellicles prepared with different glycerol contents (GL0, GL2.5, GL5, and GL10). **B**. Quantitative BET specific surface area analysis of the corresponding samples. Data are presented as mean ± SD (n = 4). Statistical significance was assessed by Student's t-test (*$P < 0.05$, ****$P < 0.0001$).

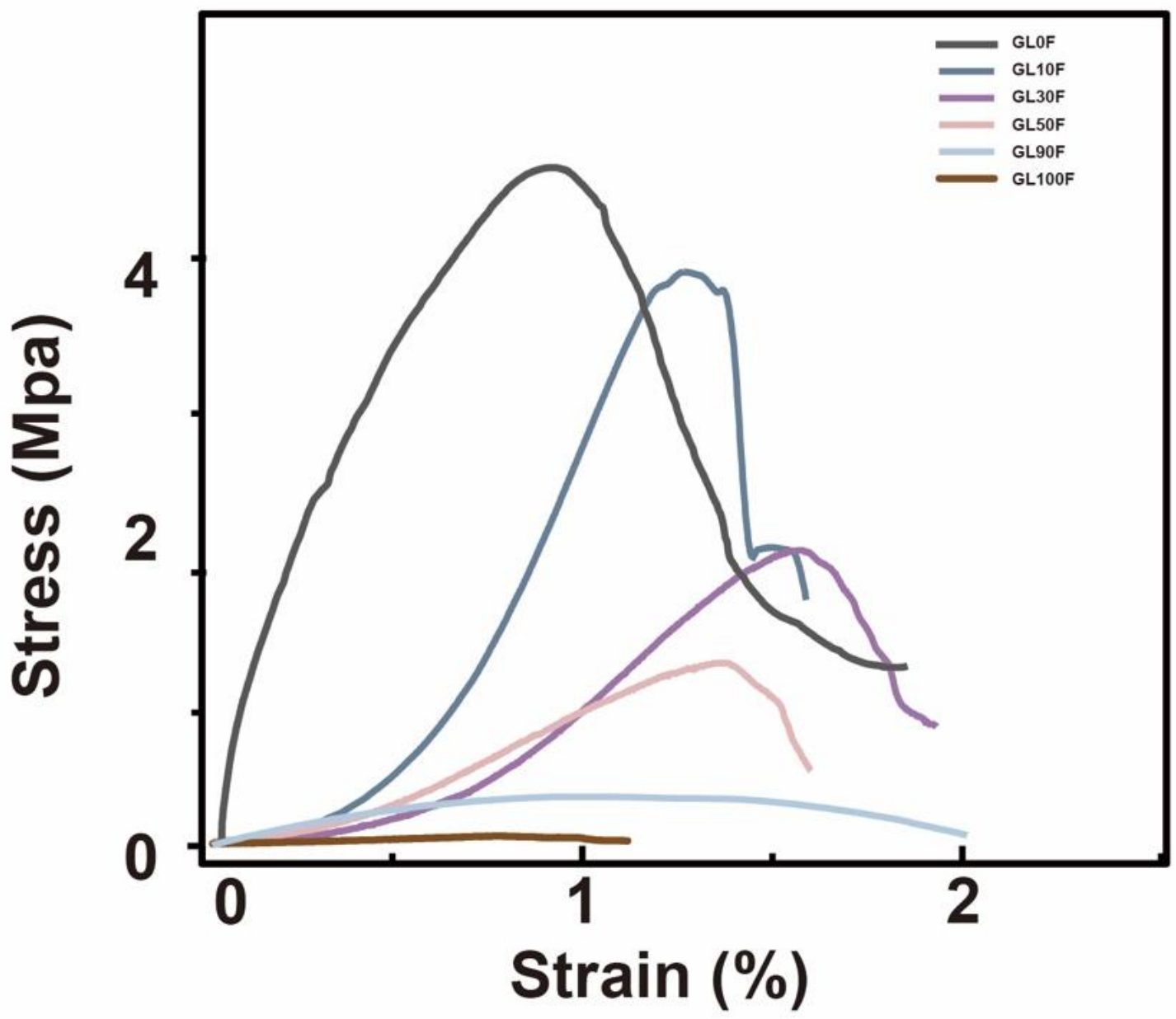

Figure S13 | Stress-strain curves of glycerol-treated pellicles. Pellicles treated with 0-100% glycerol were mechanically tested. Curves show that 10-50% glycerol enhanced ductility and elongation, while maintaining reasonable tensile strength.

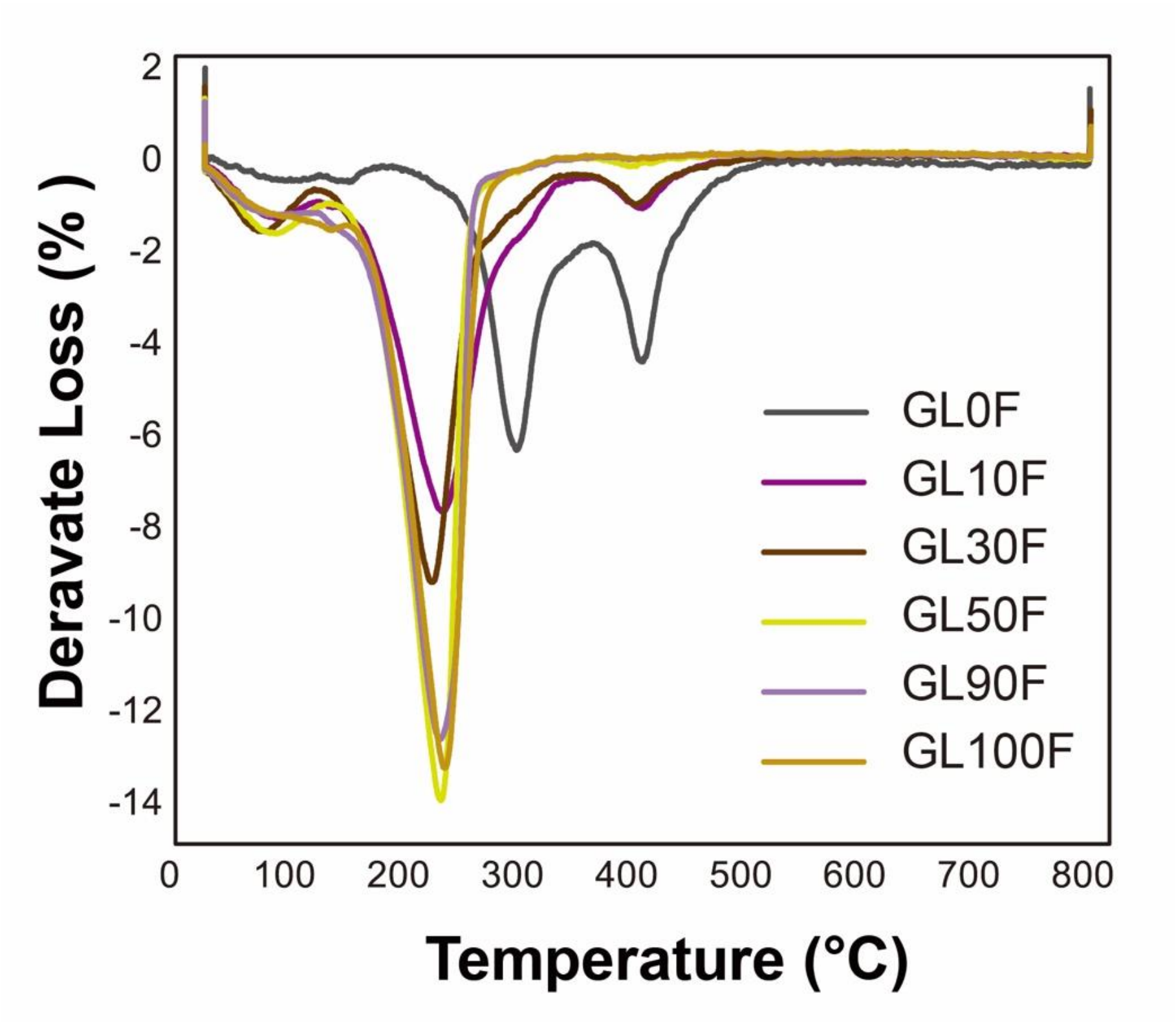

Figure S14 | DTG curves of glycerol-modified fungal pellicles. Thermal degradation rates were measured via derivative thermogravimetry (DTG) for pellicles with varying glycerol content, indicating thermal stability trends.

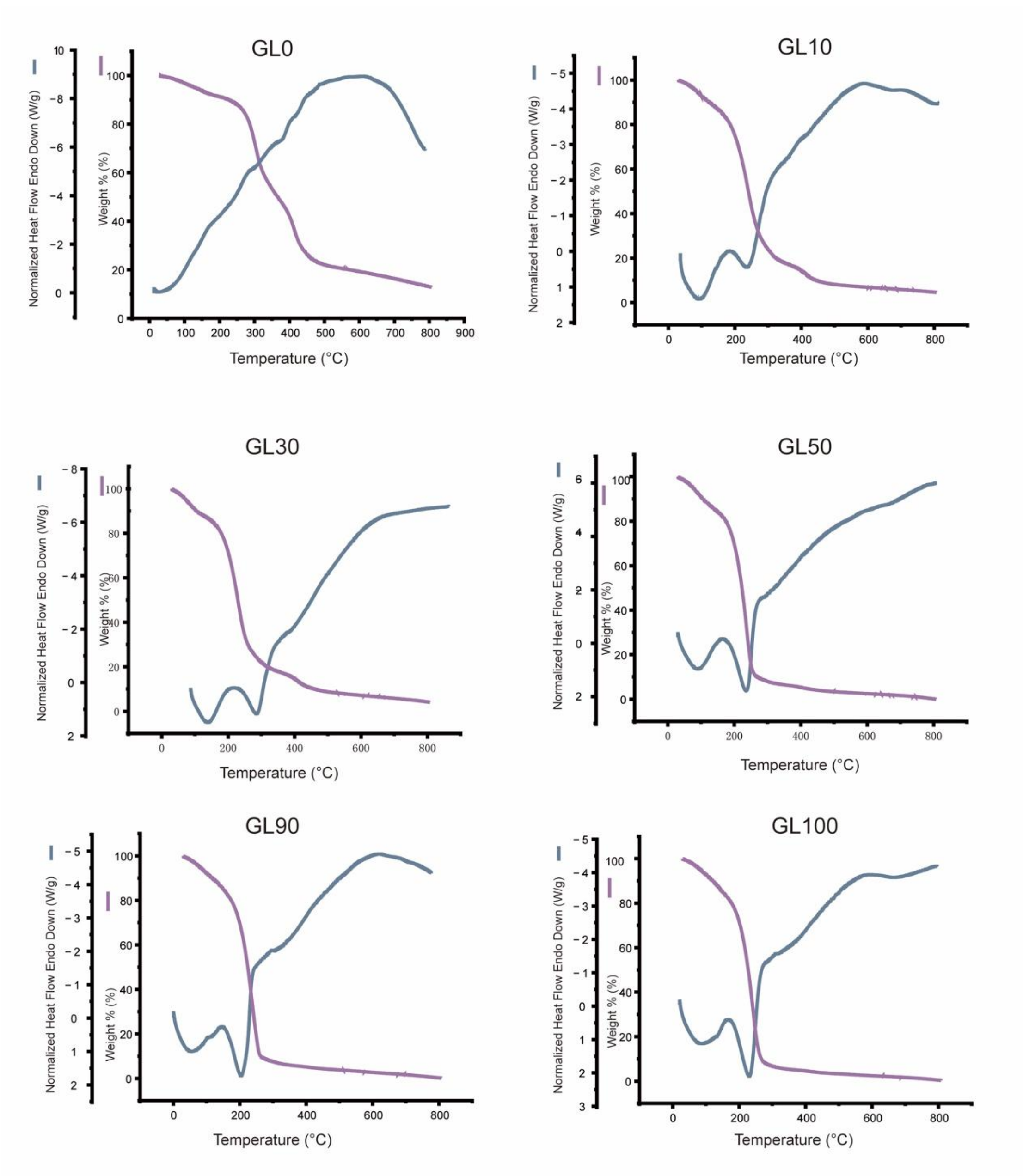

Figure S15 | TG and DSC analysis of GL-treated pellicles. Thermal decomposition and glass transition behavior were assessed using thermogravimetric (TG) and differential scanning calorimetry (DSC) analyses.

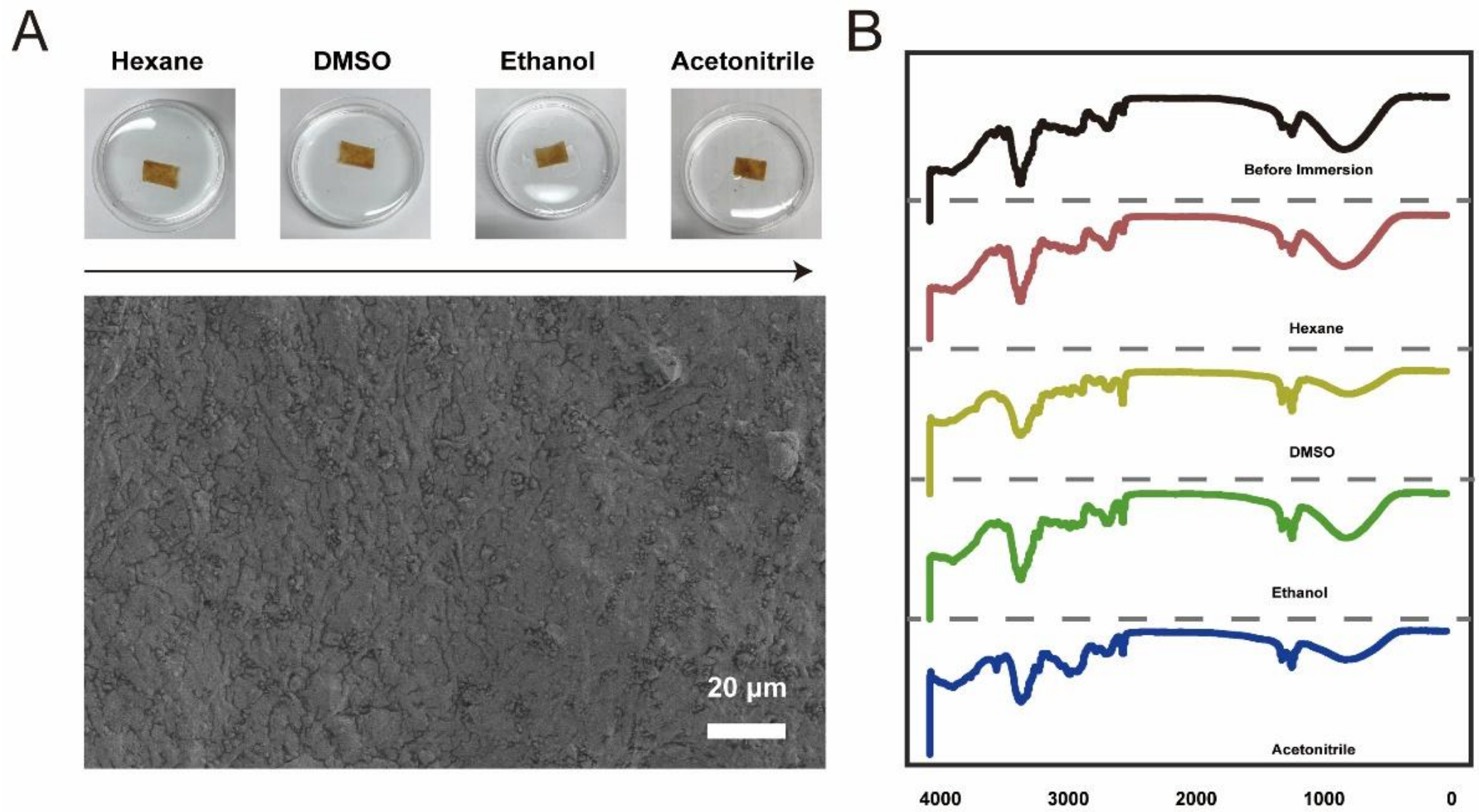

Figure S16 | Solvent stability of glycerol-treated *C. militaris* fabrics (GL10). A. Photographs of the fungal pellicle after immersion in representative organic solvents (hexane, DMSO, ethanol, and acetonitrile). A scanning electron microscopy (SEM) image of the solvent-treated pellicle is shown below, illustrating the preserved surface microstructure after solvent exposure (scale bar, 20 μm). **B**. ATR-FTIR spectra recorded before (top trace) and after (colored traces) immersion confirm no major shifts in chemical composition.

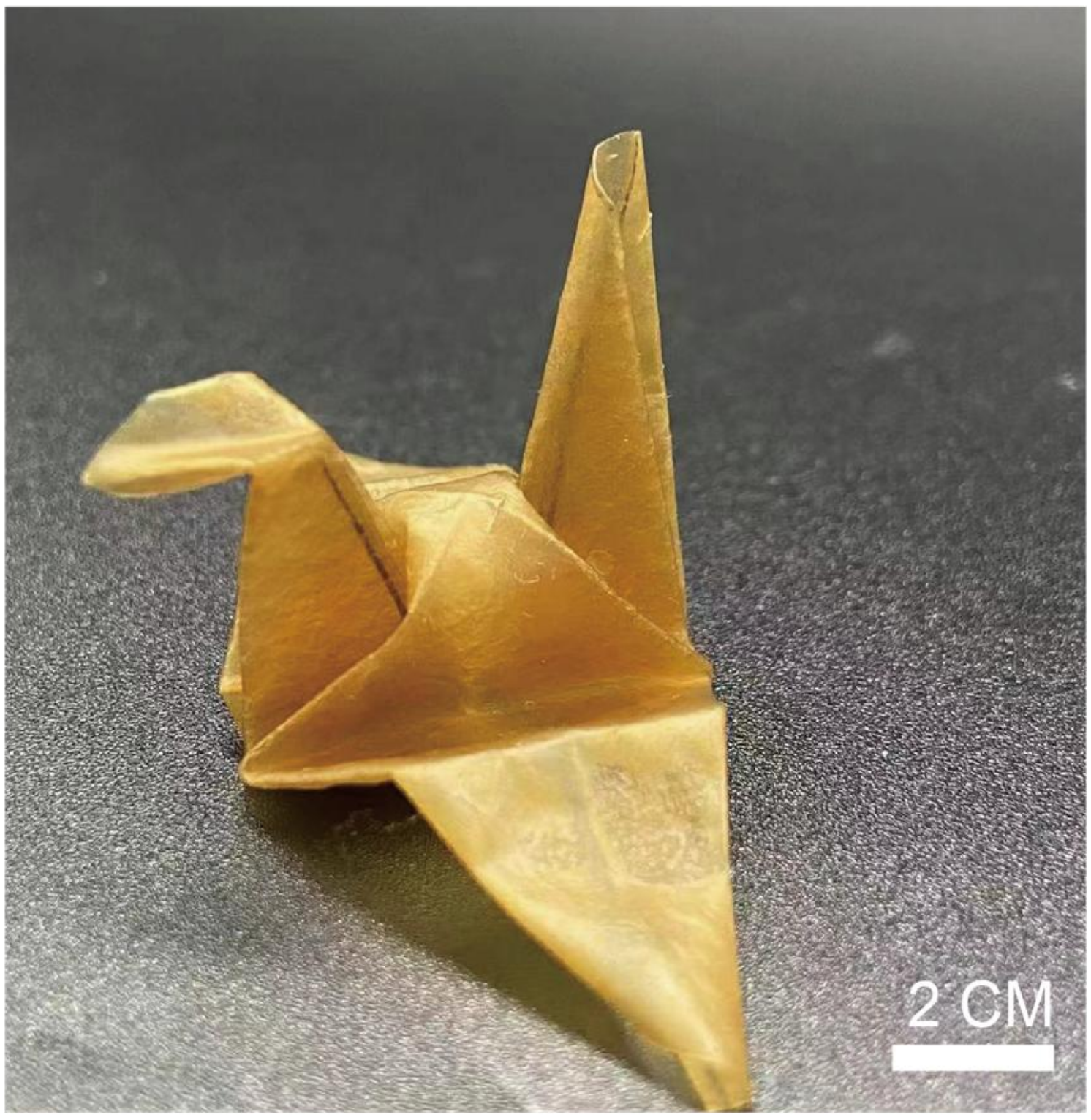

Figure S17 | Mechanical flexibility of GL10 pellicles. An origami crane was folded from a GL10 sheet, demonstrating sufficient flexibility and crack resistance post-plasticization.

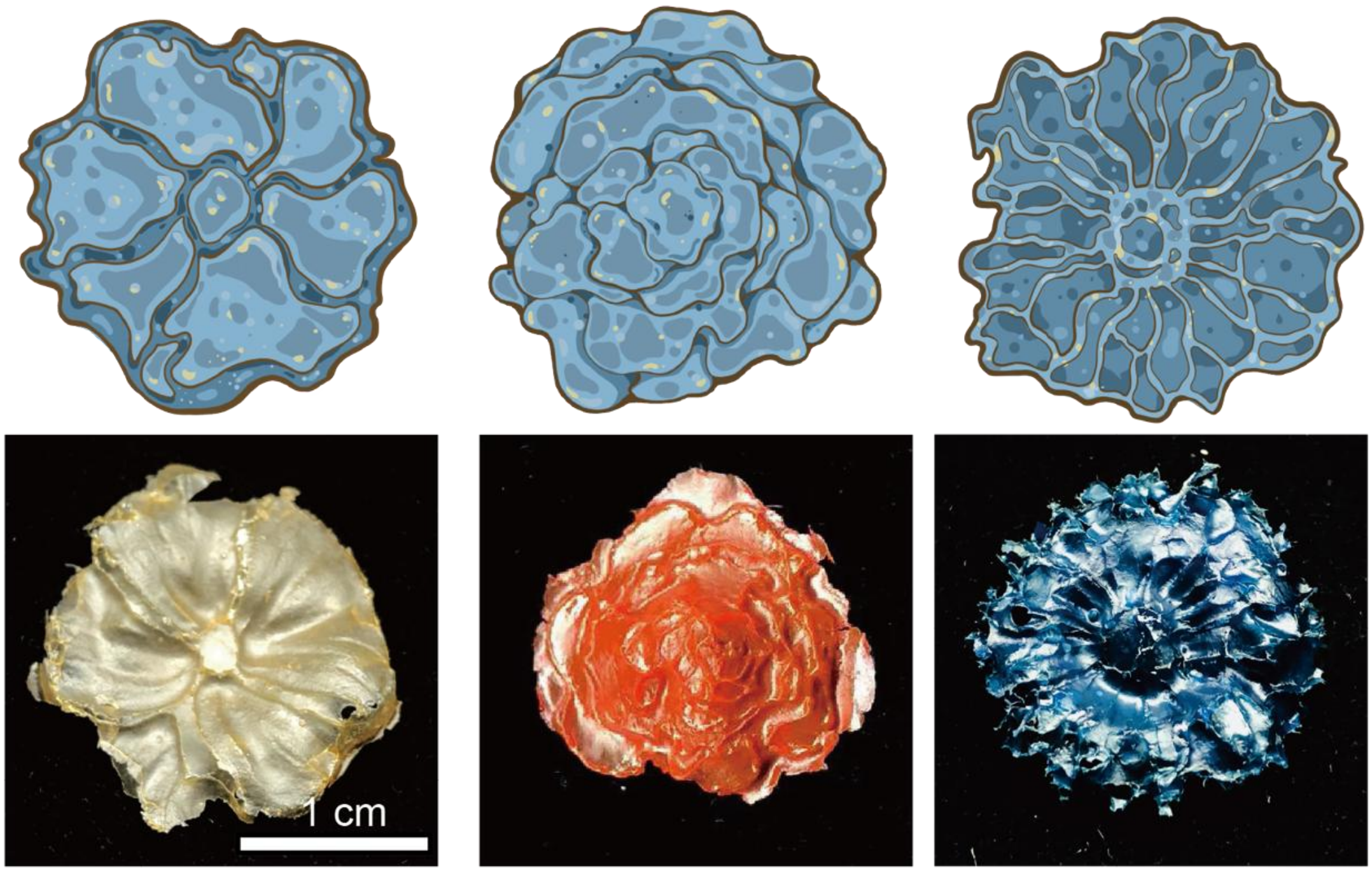

Figure S18 | 3D molding capability of GL10 sheets. Examples of curved and folded geometries fabricated using semi-wet GL10 pellicles, highlighting shape adaptability.

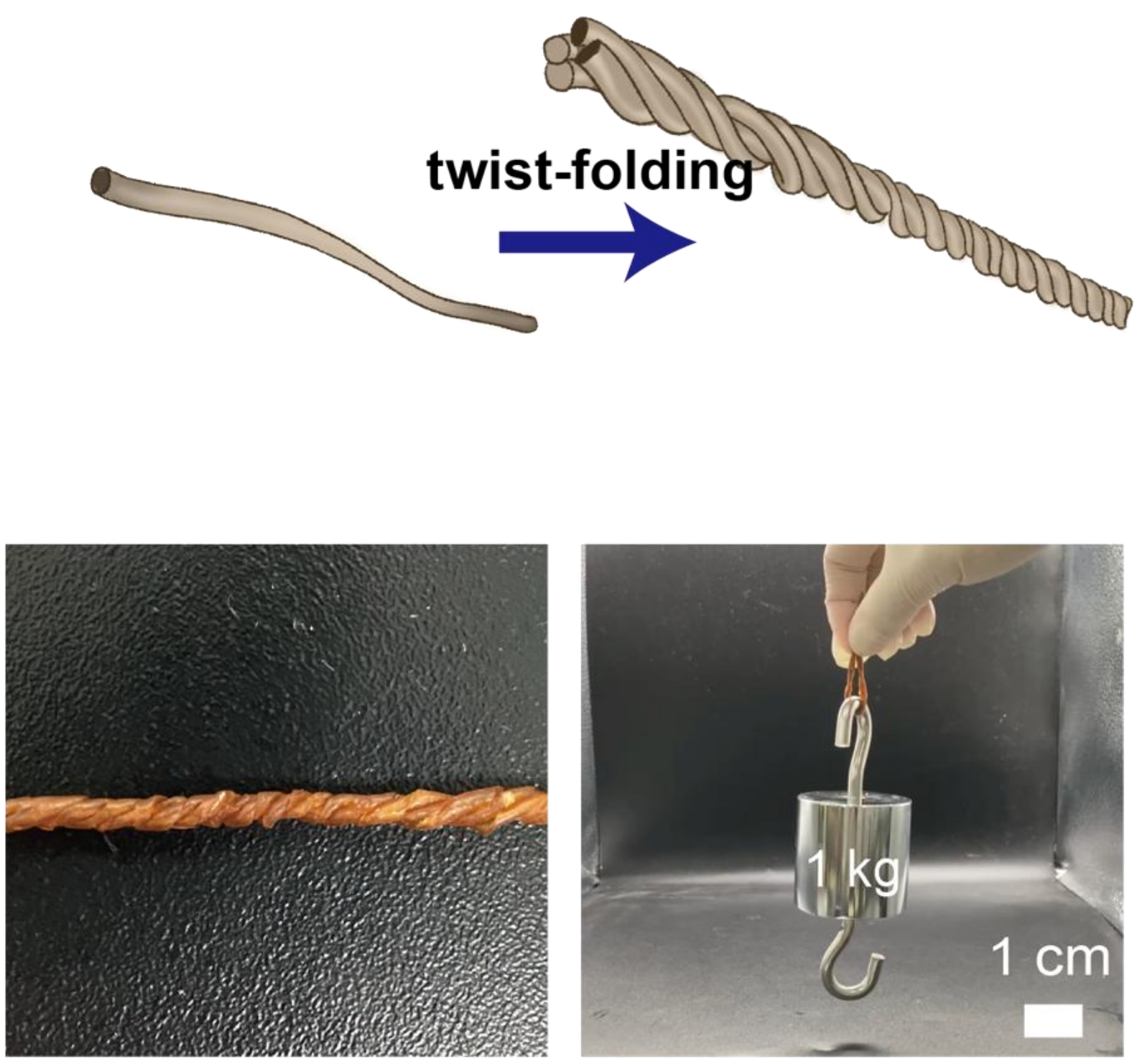

Figure S19 | Load-bearing demonstration with twisted GL10 strips. Four twisted GL10 strips (1 mm wide, 0.18 cm thick) were braided into a rope and used to lift a 1 kg weight.

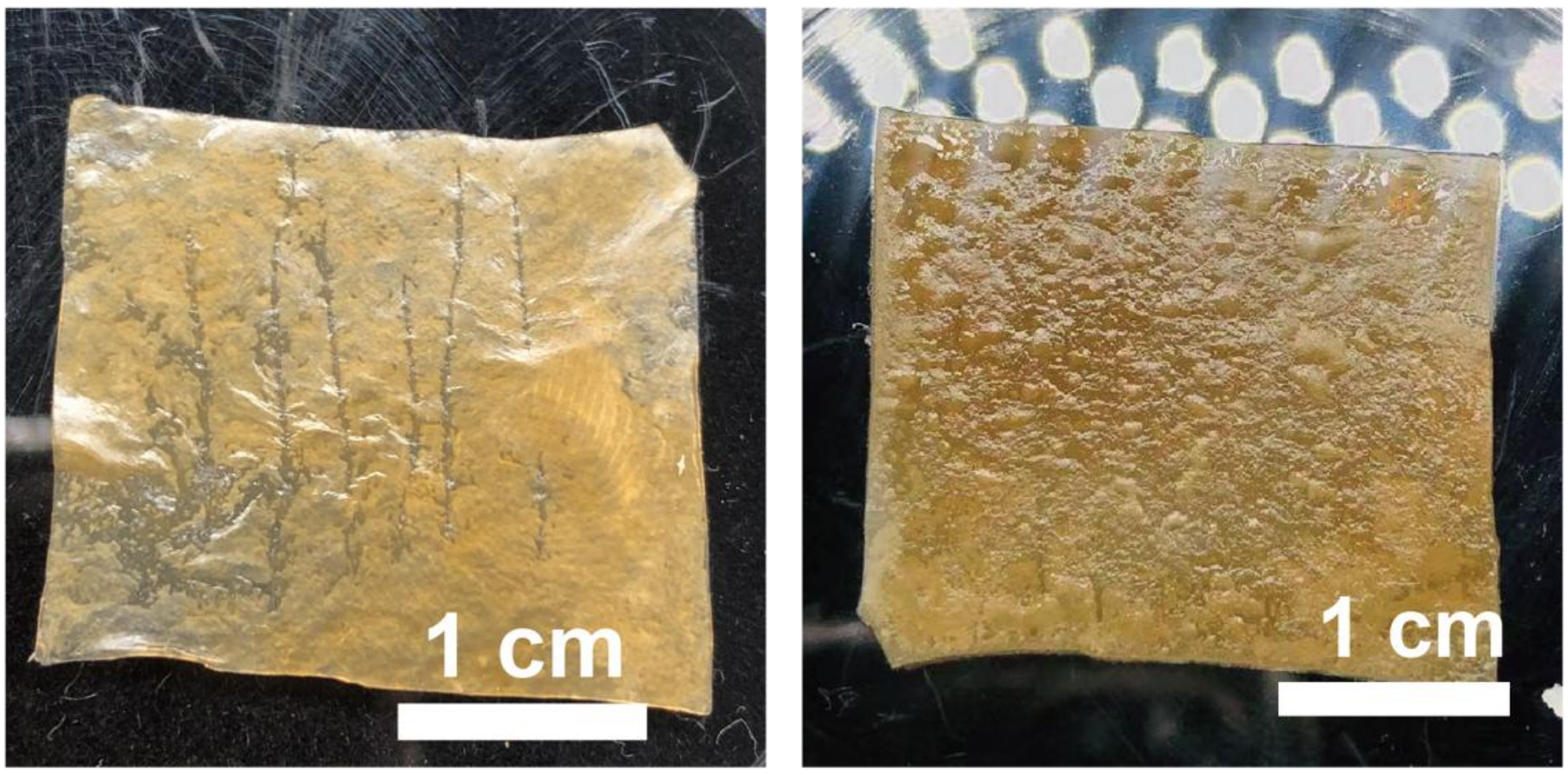

Figure S20 | Self-healing of GL10 pellicles upon water treatment. Cut GL10 pellicles were rejoined by applying water to the incision, followed by drying. The surface reformed into a continuous film.

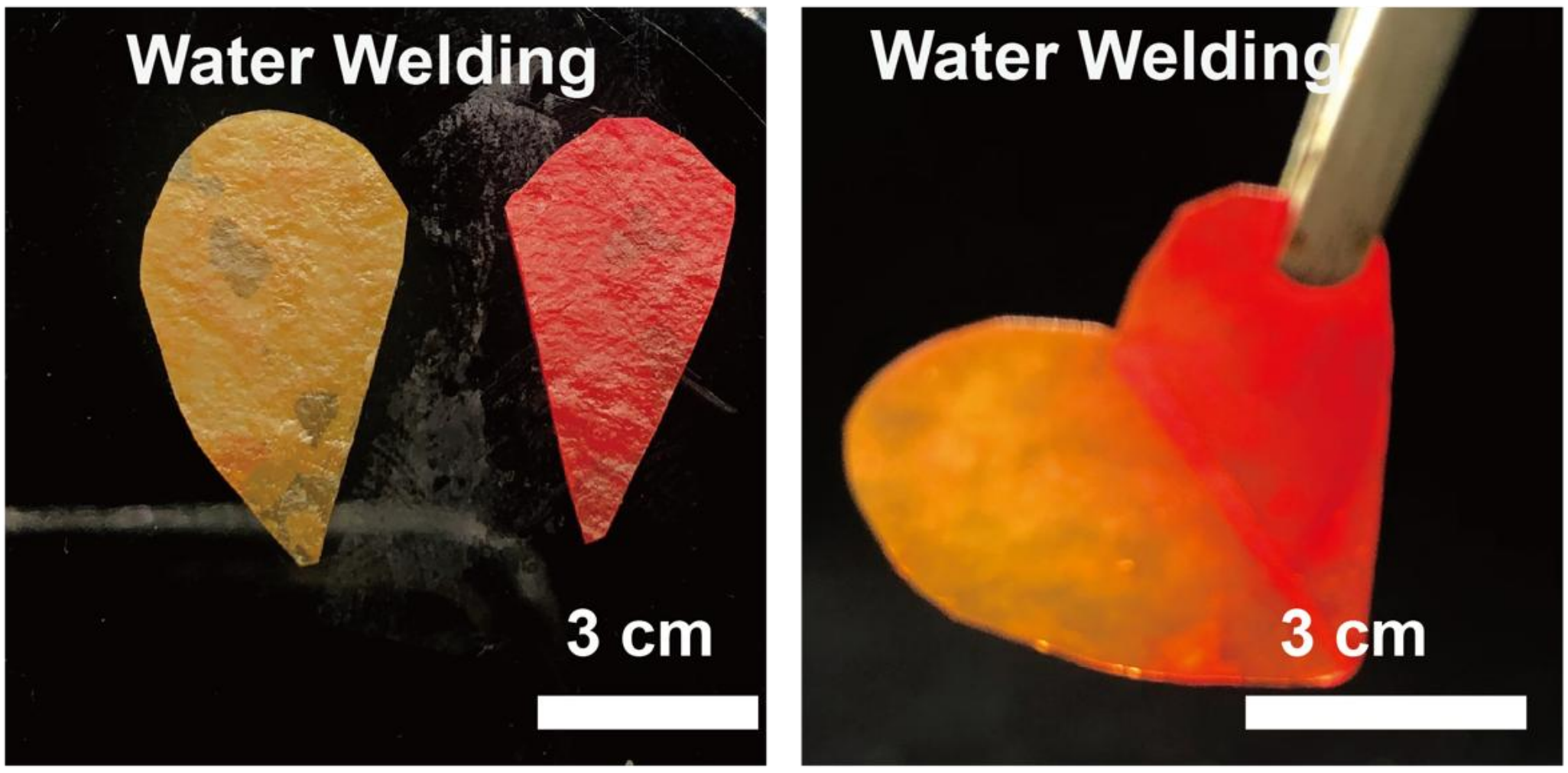

Figure S21 | Seamless fusion of GL10 pellicles using water. Two lateral GL10 pieces were adhered edge-to-edge using water as a bio-glue, resulting in strong, seamless integration.

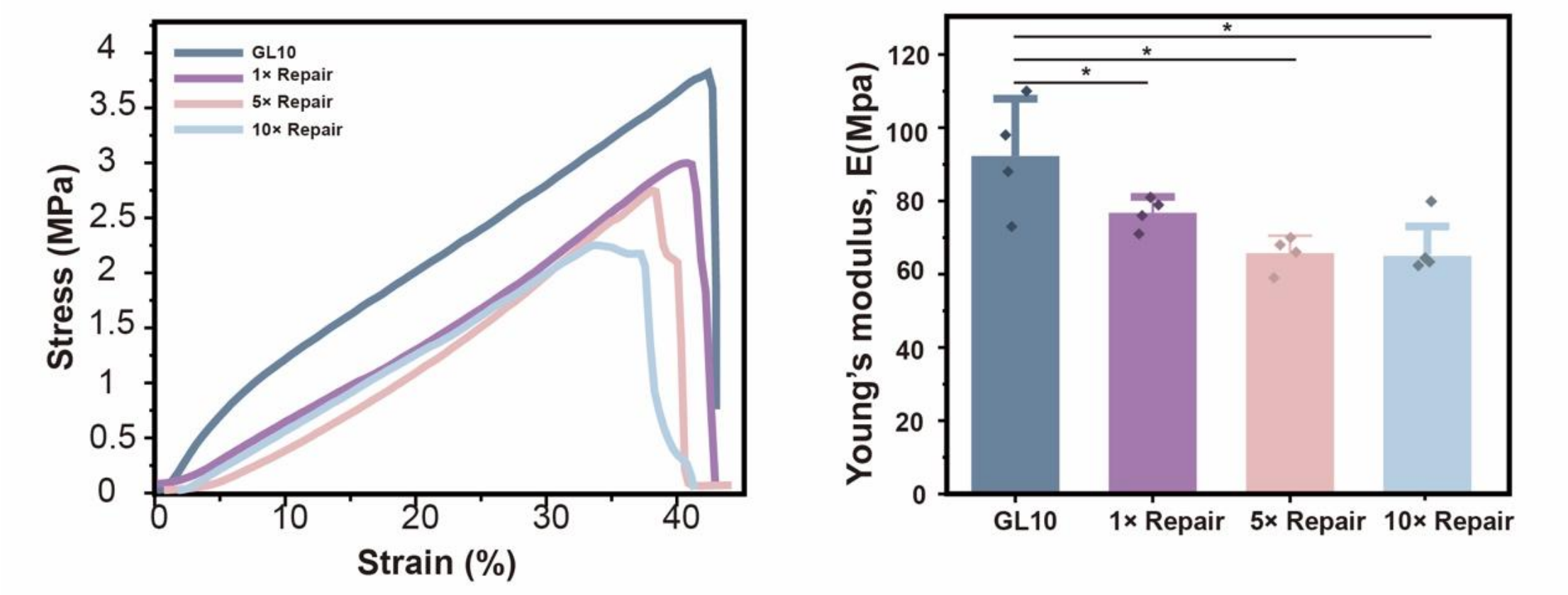

Figure S22 | Mechanical performance of the material after repeated water-enabled repair cycles. Left: Representative stress-strain curves of the pristine sample (GL10) and samples subjected to 1, 5, and 10 repair cycles. Right: Young's modulus of the pristine and repaired samples, quantified from the initial linear region of the stress-strain curves. Data are presented as mean ± s.d. (n = 4). Repair in this assay mainly depends on reversible hydrogen bonding and chain interdiffusion after water addition and drying, resulting in approximately 68% stiffness retention after 10 cycles. Statistical significance was determined by Student's t-test (*P < 0.05).

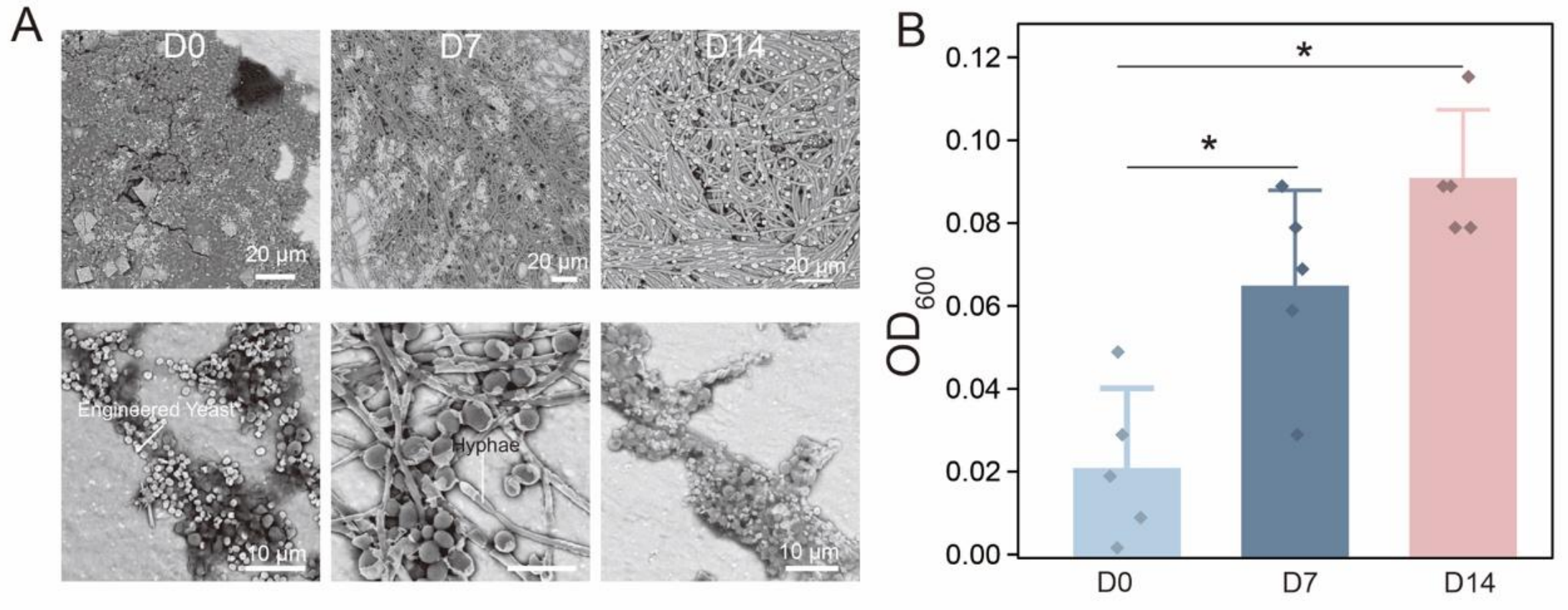

Figure S23 | Qualitative assessment of CBD-mediated yeast attachment stability under prolonged immersion. A. SEM images of yeast-mycelium composites after immersion in water for 0, 7, and 14 days (D0, D7, D14), showing that engineered yeast cells remain closely associated with the fungal hyphal network over time. **B**. OD_{600} of the immersion supernatant collected at D0, D7, and D14. Data are presented as mean ± s.d. (n = 5). Statistical significance was assessed using Student's t-tests (*P < 0.05).

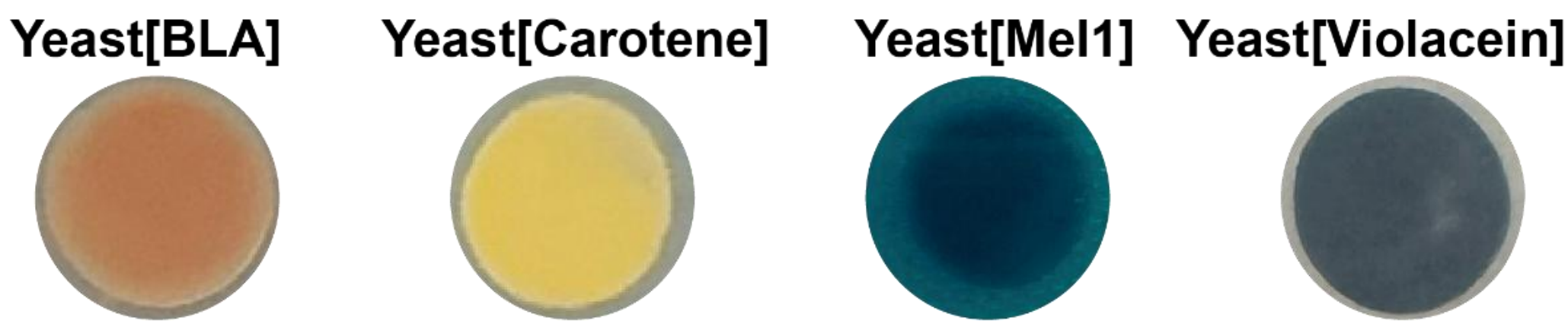

Figure S24 | Color tuning via different yeast strains. Tunable pigmentation generated by engineered yeast strains, ranging in color from red to purple on the pellicle surface.

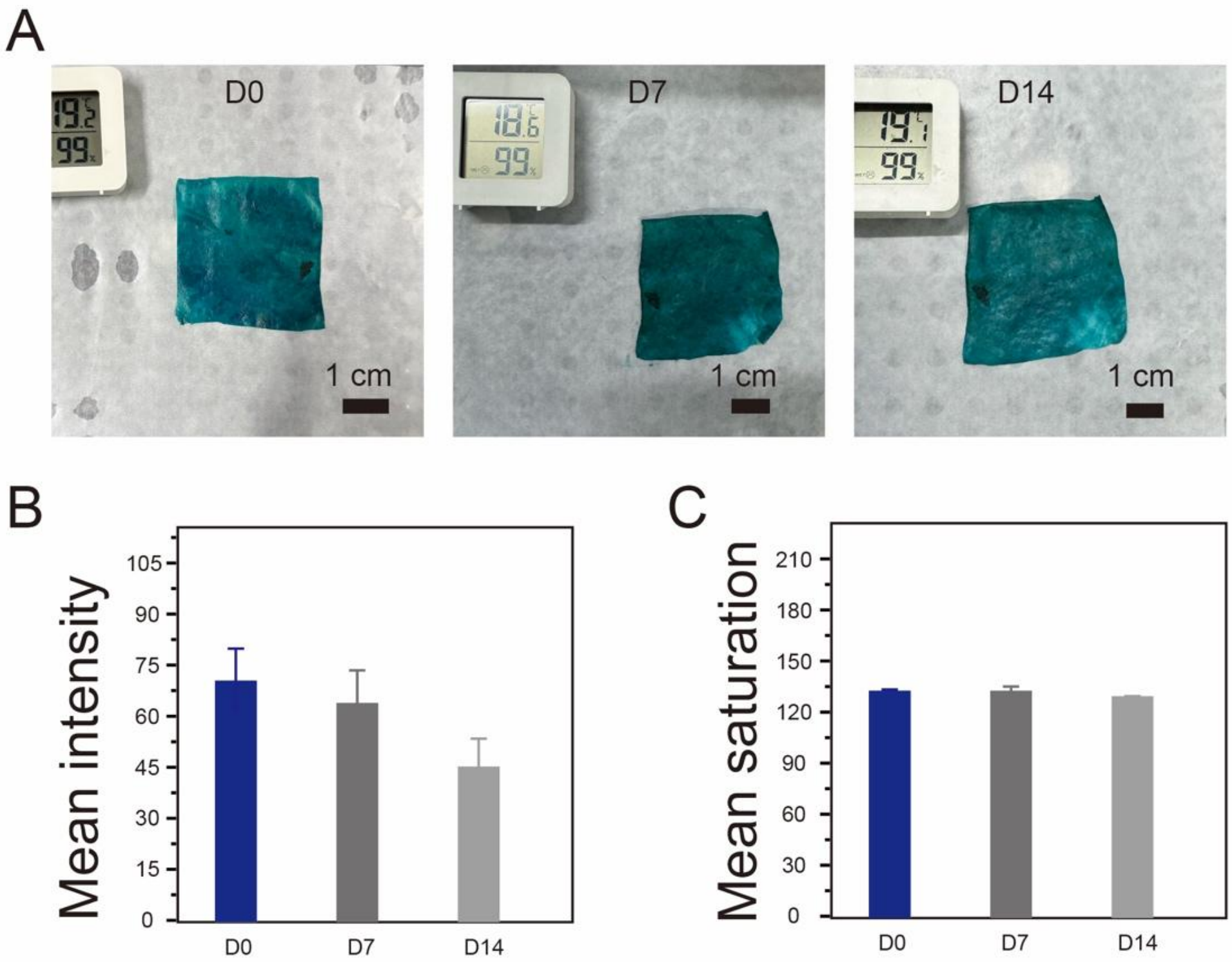

Figure S25 | Temporal stability of post-growth pigmentation in living fungal textiles. A. Representative photographs of pigmented *C. militaris* textiles at day 0 (D0), day 7 (D7), and day 14 (D14) after post-growth functionalization with pigment-producing *S. cerevisiae*, stored under controlled high-humidity conditions (~99% RH, ~19 °C). Scale bars, 1 cm. **B**. Quantitative analysis of mean grayscale intensity of the pigmented textiles over time. **C**. Quantitative analysis of mean color saturation over the same period. Data represent mean ± s.d. from three independent regions of interest.

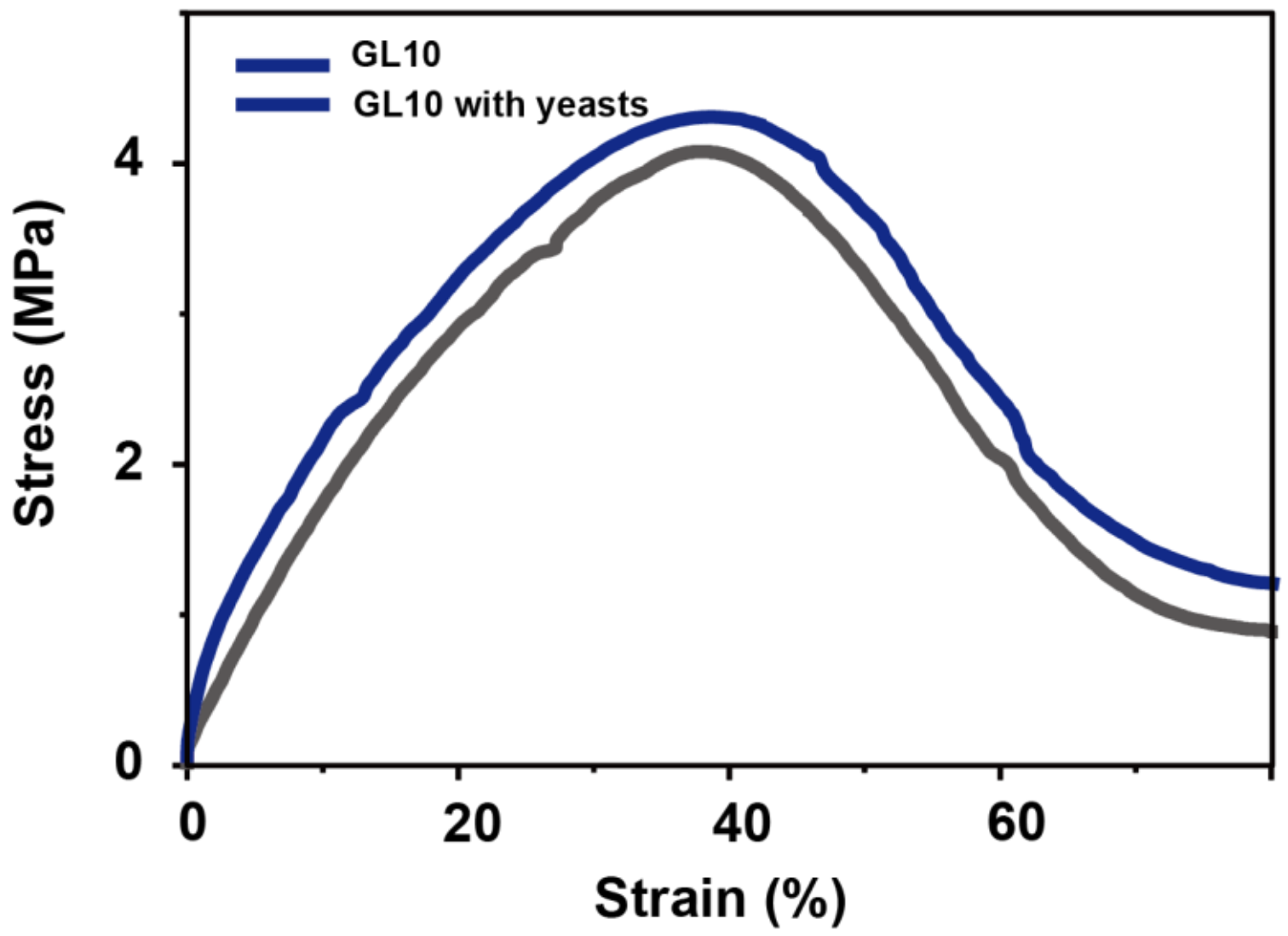

Figure S26 | Mechanical performance of pellicles after yeast incorporation. Stress-strain testing of GL10 pellicles post-yeast colonization confirms minimal change in mechanical integrity.

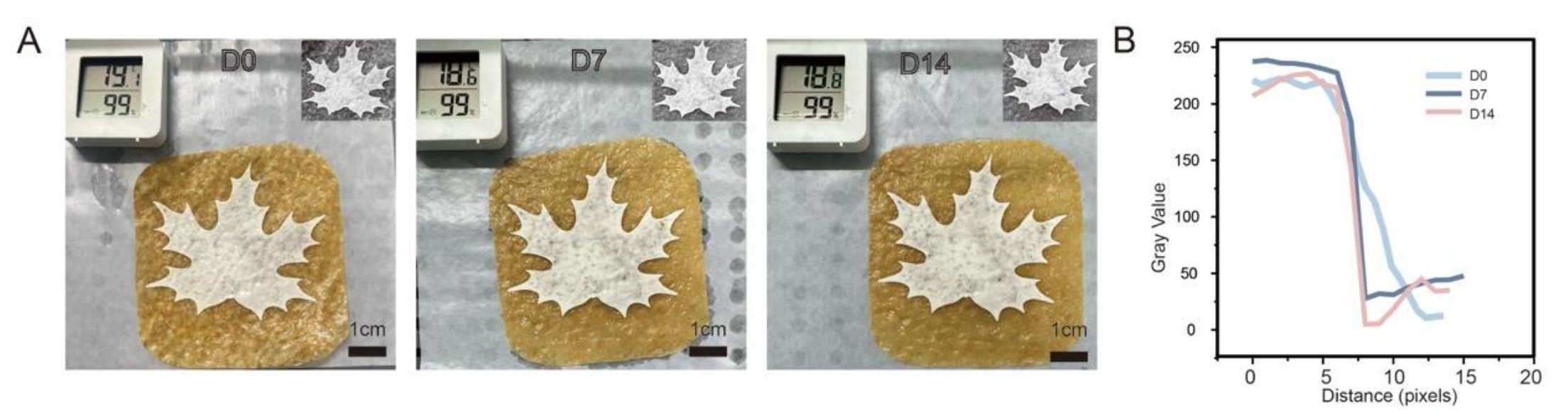

Figure S27 | Time-dependent edge expansion of aerial hyphae in fungal materials. **A**. Representative images of fungal materials at day 0 (D0), day 7 (D7), and day 14 (D14) under identical high-humidity storage conditions (~99% relative humidity, ~19 °C). **B**. Grayscale intensity profiles extracted along lines perpendicular to the material edge using ImageJ.

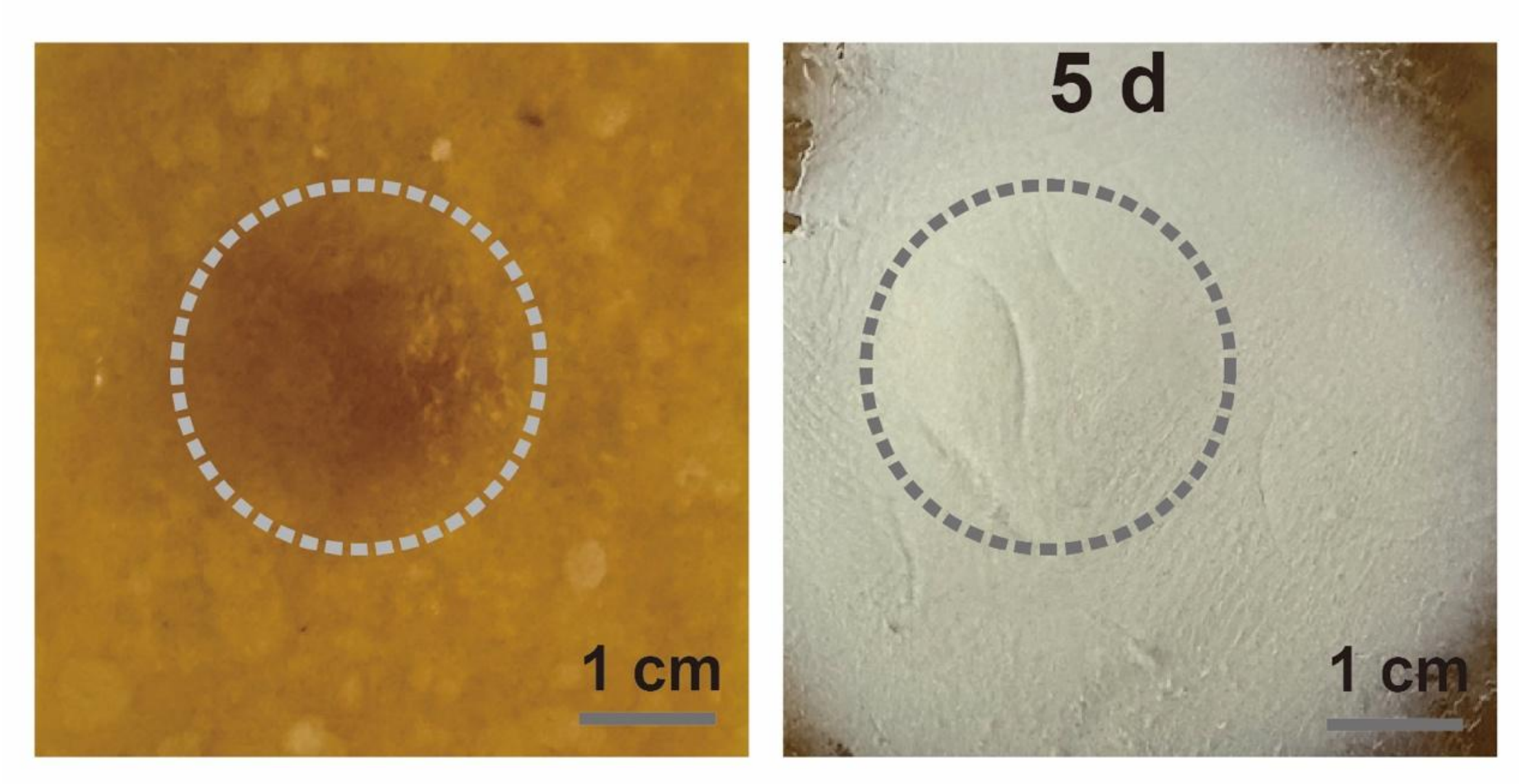

Figure S28 | Representative photographs of a fungal pellicle showing localized aerial hyphal growth without the use of any external physical barrier.

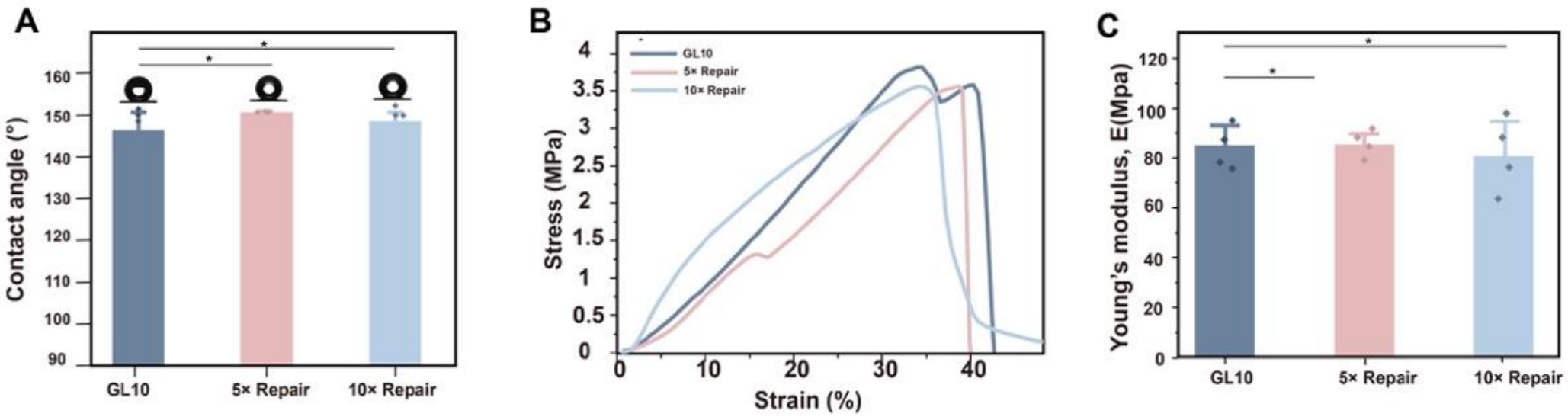

Figure S29 | Hydrophobic and mechanical performance of fungal materials after pellet-mediated repeated self-repair cycles. A. Static water contact angle of the pristine sample (GL10) and samples subjected to 5 and 10 self-repair cycles (n=3 independent replicates). **B**. Representative stress-strain curves of the pristine material and samples after 5 and 10 repair cycles. **C**. Young's modulus quantified from the initial linear region of the stress-strain curves (n = 4 independent replicates). In this assay, damaged regions were repaired by introducing fresh mycelial pellets followed by nutrient-induced aerial hyphae regrowth, thereby restoring both structural continuity and surface functionality. Statistical significance was determined by Student's t-test (*P < 0.05).

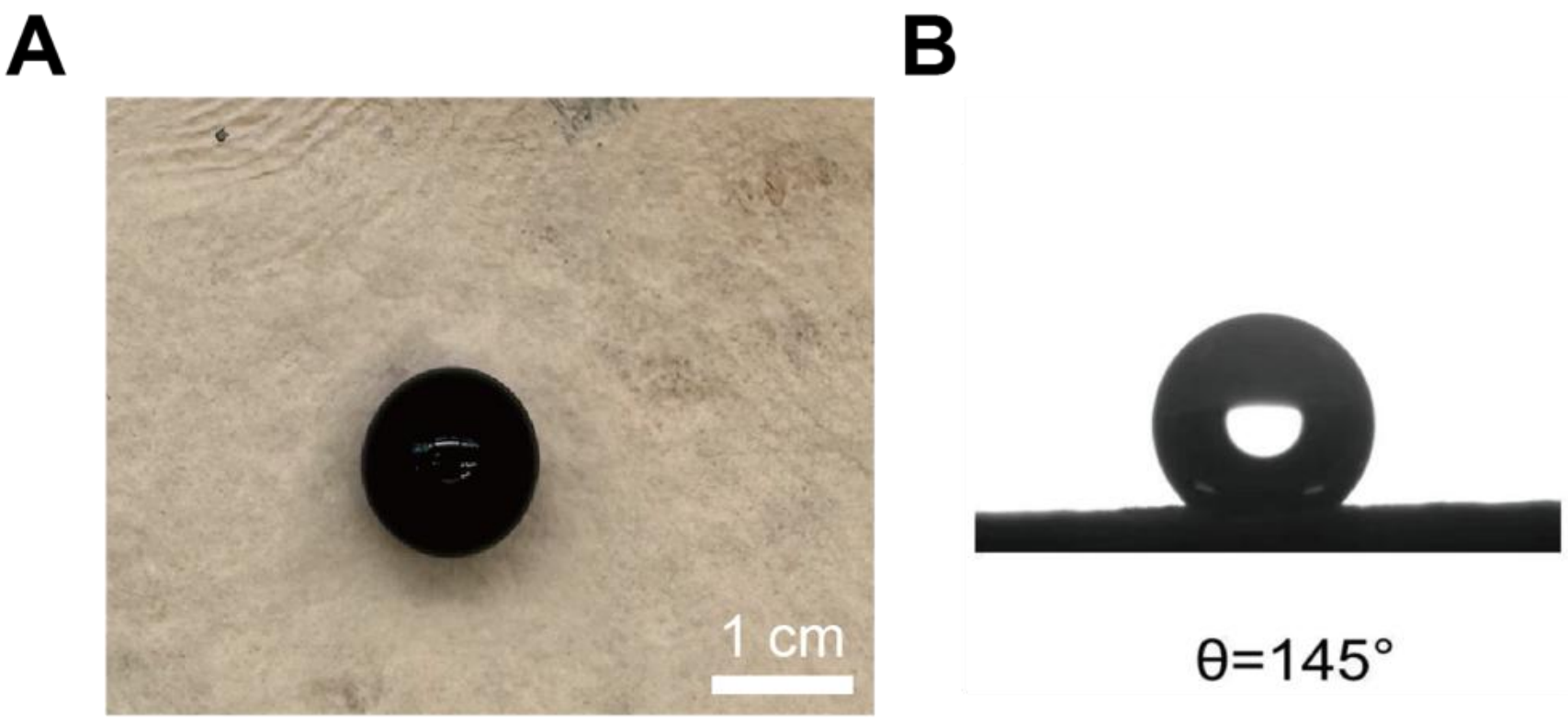

Figure S30 | Contact angle measurement after aerial hypha formation. A, B. Formation of aerial hyphae on GL10 increased water contact angle to ~145°, indicating improved surface hydrophobicity.

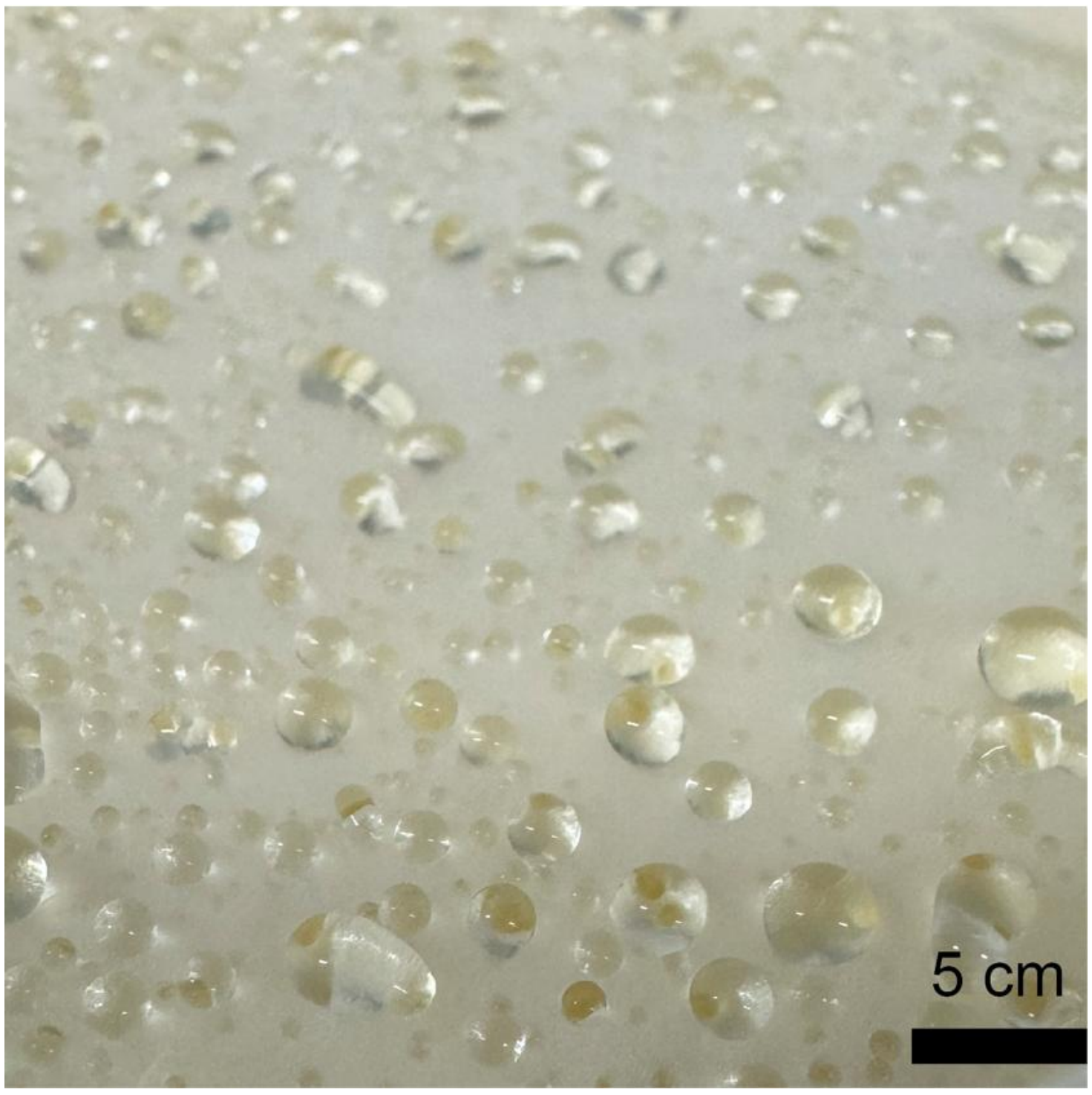

Figure S31 | Antifouling behavior of aerial hypha-coated pellicles. Close-up view of water droplets rolling off the aerial hypha-covered GL10 pellicle surface, highlighting antifouling or self-cleaning behavior.

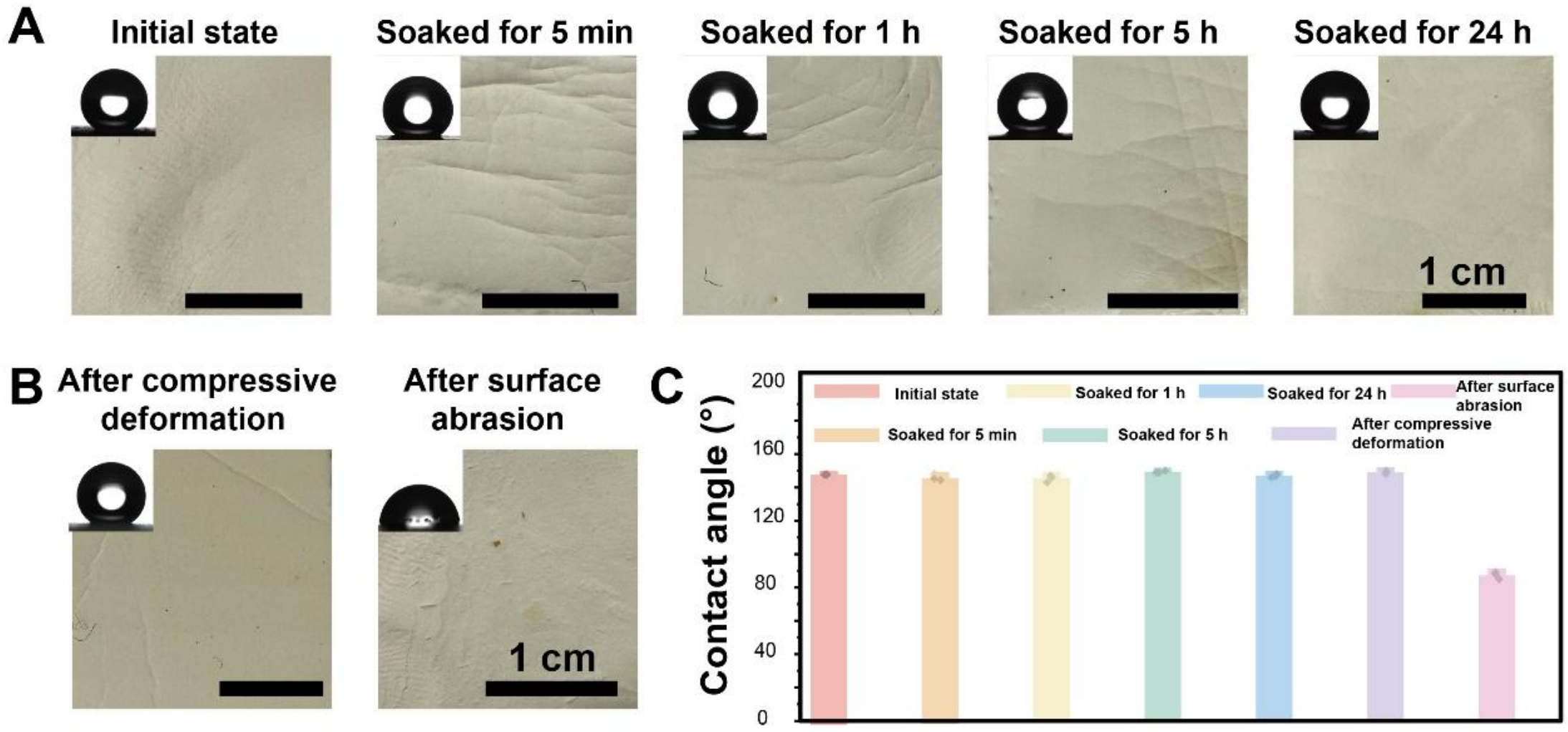

Figure S32 | Durability of surface hydrophobicity under wetting exposure and mild mechanical perturbations. A. Representative static water contact angle (WCA) images of initial state, soaking for 5 min, 1 h, 5 h, or 24 h, after compressive deformation, and after surface abrasion, followed by air-drying. **B.** Representative WCA images of samples subjected to mechanical compression and mild surface abrasion. **C.** Quantification of static WCA under different treatments. Data are presented as mean ± s.d. (n = 3 independent samples).

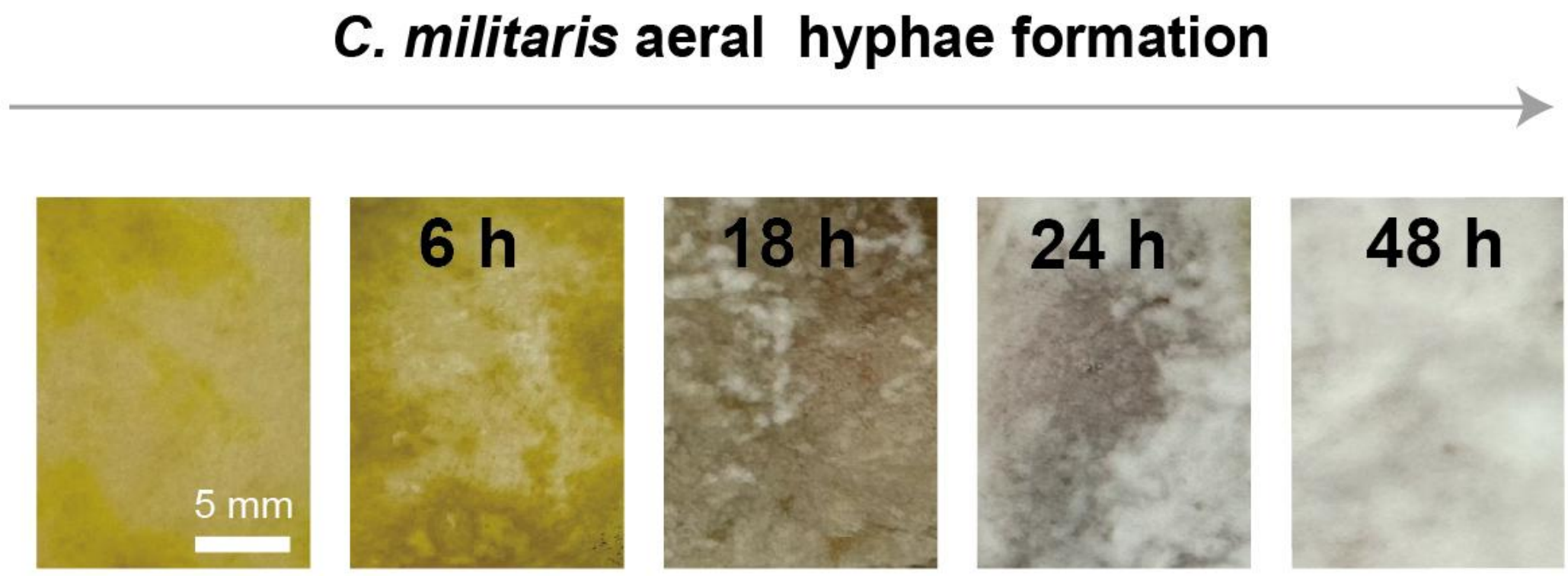

Figure S33 | Microscopy of aerial hyphae induced on GL10. Representative optical images showing aerial hyphae formation on a fungal pellicle recorded from the same culture plate at different time points (0 h, 6 h, 18 h, 24 h, and 48 h) after nutrient stimulation. The images illustrate the progressive development and expansion of aerial hyphae across the pellicle surface. Scale bar, 5 mm.

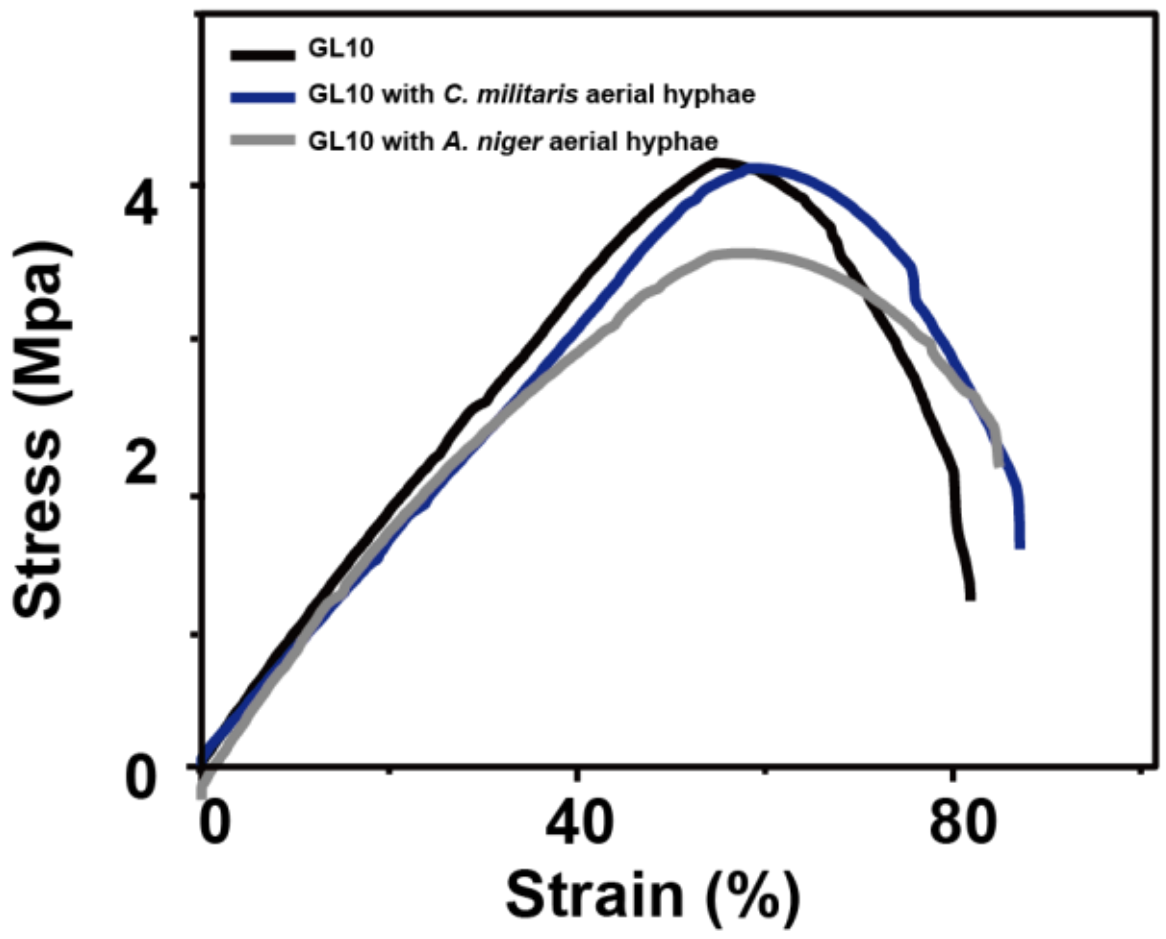

Figure S34 | Stress-strain comparison before and after aerial hypha growth. Mechanical testing reveals that hypha formation does not compromise tensile strength or elongation of GL10 pellicles.

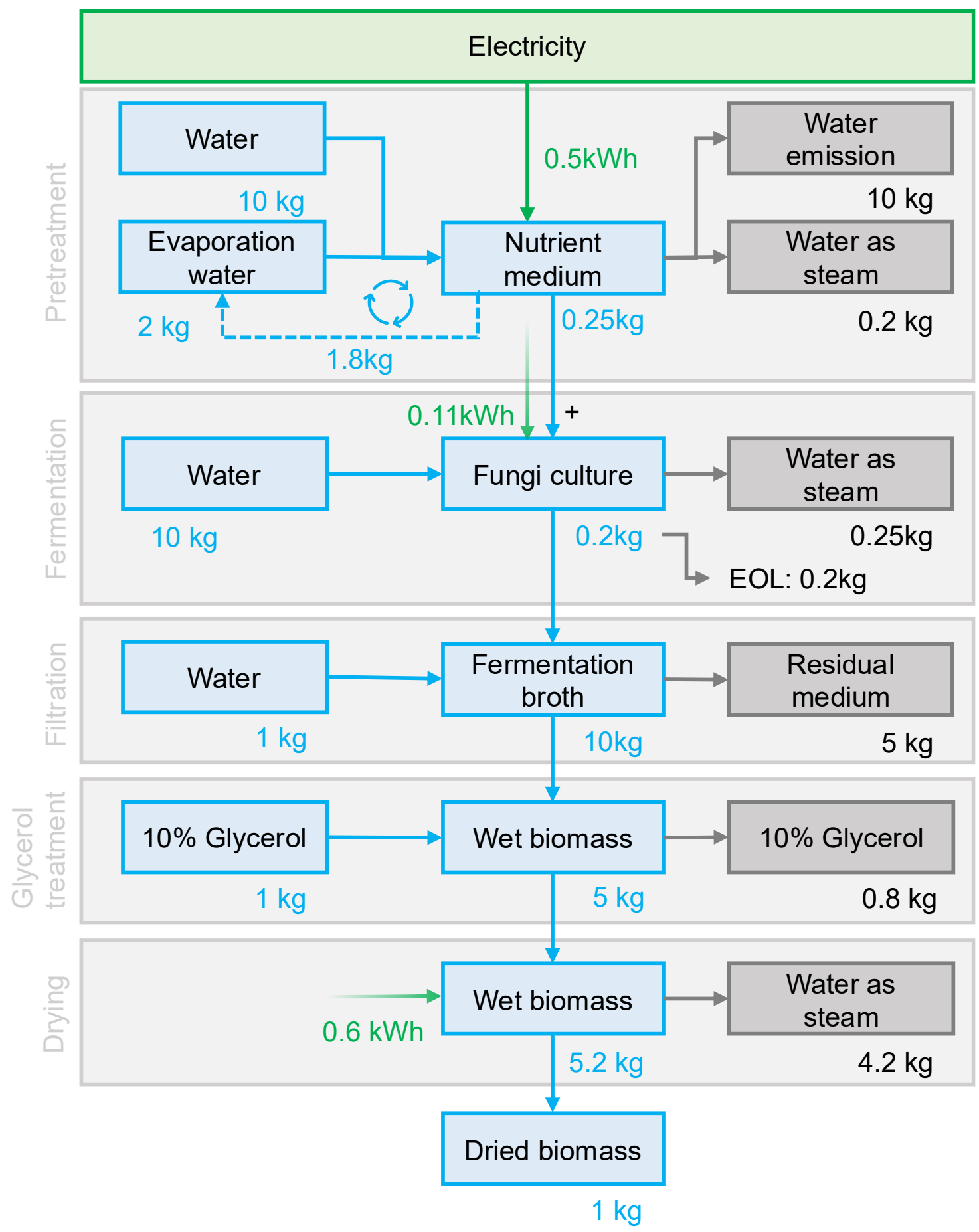

Figure S35 | Simplified cradle-to-gate process flow for producing 1 kg of dried *C. militaris* biomass used in the LCA. The diagram illustrates five main stages—pretreatment, fermentation, filtration, glycerol treatment, and drying—along with associated mass flows (blue boxes) and energy use (green text). Water and nutrient medium inputs, as well as electricity consumption, are traced through each stage, while outputs include evaporated water, residual medium, and the final dried biomass. This schematic captures the system boundary applied in the life cycle assessment and highlights key process streams relevant to the environmental impact analysis.

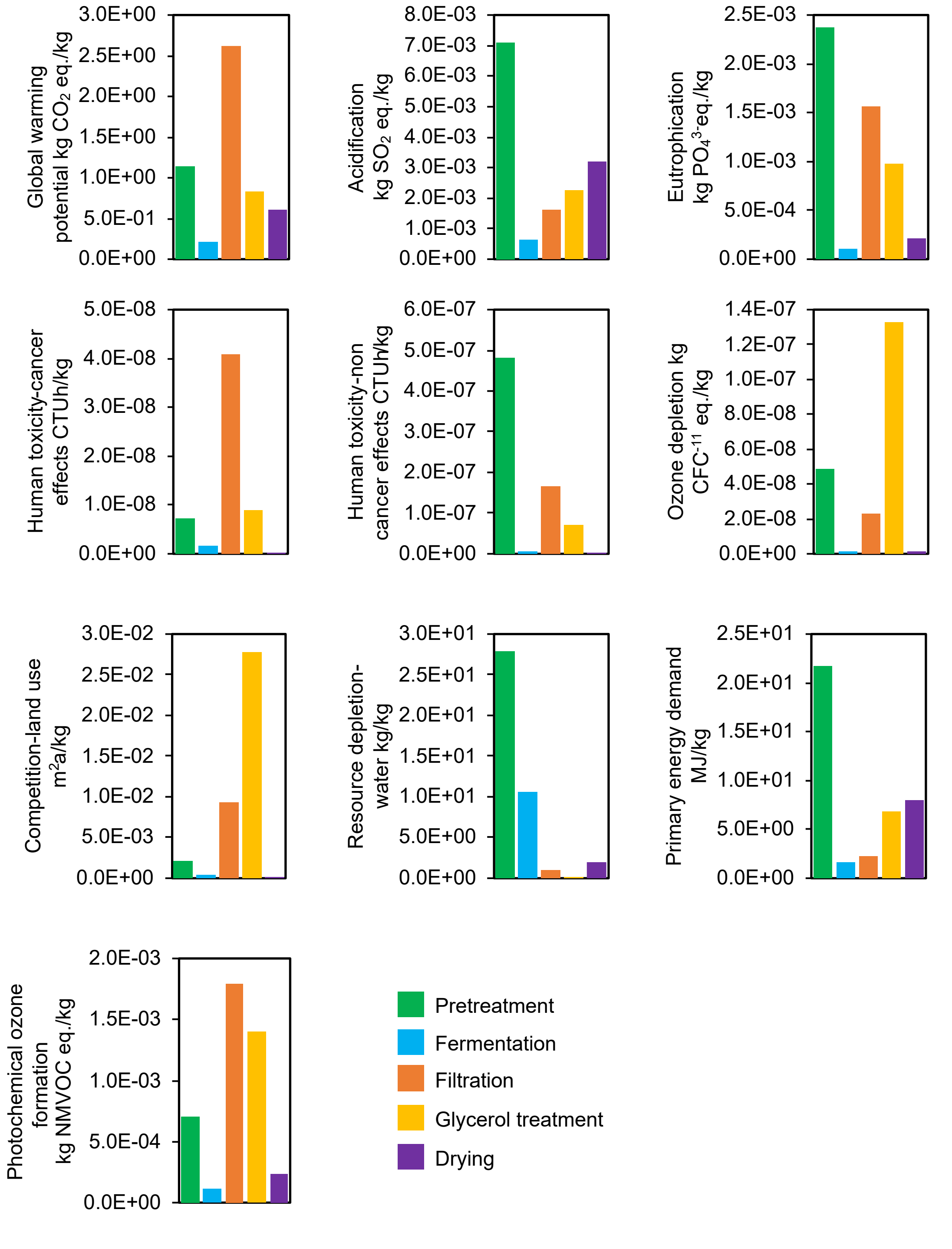

Figure S36 | LCA-derived environmental impact indicators. Key environmental impact indicators (e.g., GWP, AP, EP) from the LCA analysis of producing 1 kg dried fungal biomass, subdivided by process stages (S1-S5).

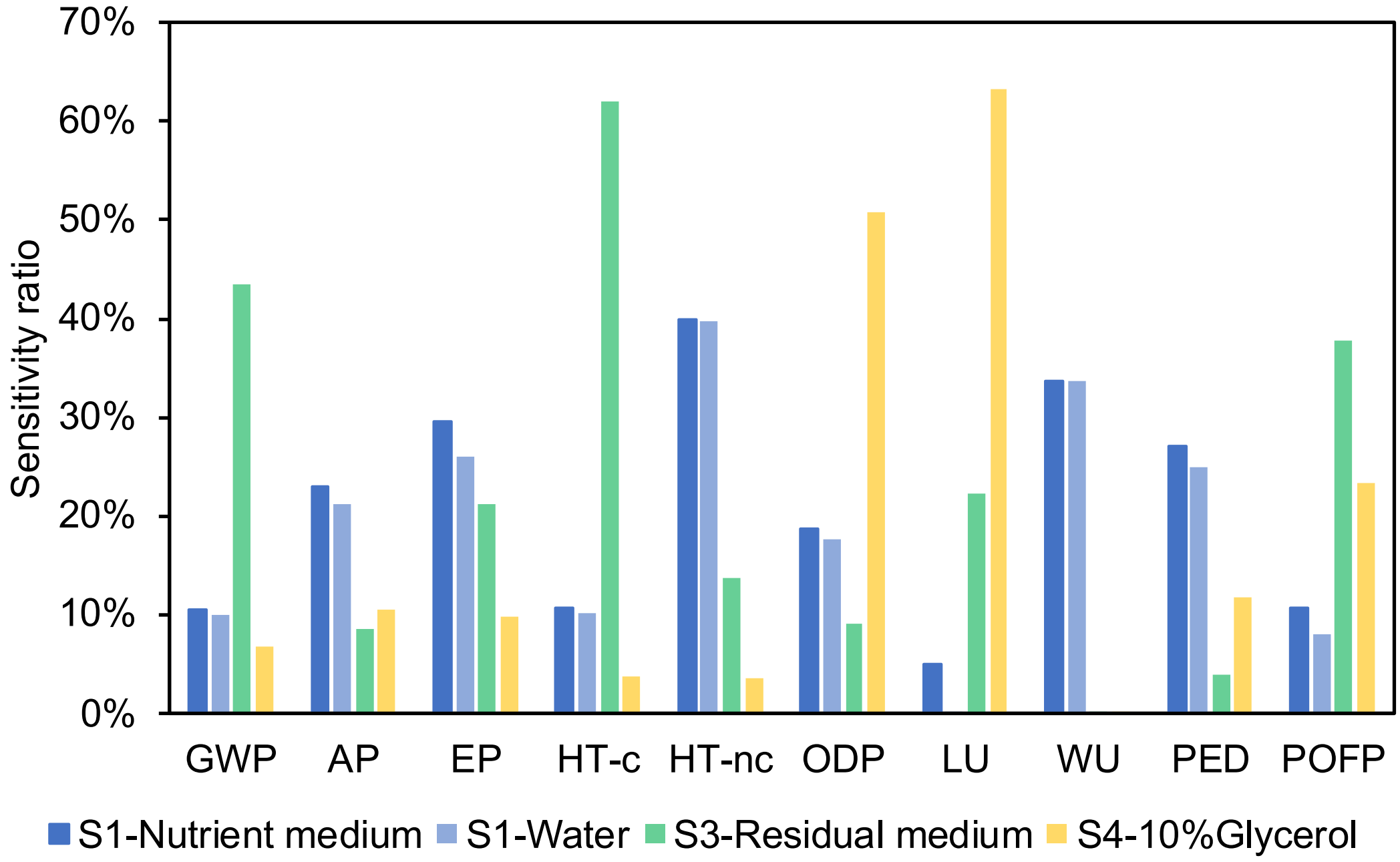

Figure S37 | Sensitivity analysis of four LCA parameters. Sensitivity ratio of four primary driving factors in the LCA impact categories, illustrating how variations in media composition, water usage, glycerol sourcing, and energy inputs shift overall environmental burdens.

Table S1 Different polysaccharide–glycerol systems constructed

System	Glycerol content %	$C_8H_{13}O_5$ /mol	$C_6H_{10}O_5$ /mol	$C_3H_8O_3$ /mol	Degree of polymerization	Total atoms
S0	0	47	24	0	10	15742
S1	10	47	24	136	10	17646
S2	30	47	24	406	10	21426
S3	50	47	24	680	10	25262
S4	90	47	24	1224	10	32878
S5	100	47	24	1360	10	34782

Table S2 Water contact angle (WCA) of fungi-based fabrics. From a-f: a represents untreated fungi based fabric ; b-f represent fungi based fabrics treated with different concentrations of glycerol (b=10%, c=30%, d=50%, e=90%, and f=100%), respectively.

	a	b	c	d	e	f
WCA (°)	61 ± 2	51 ± 4	40 ± 3	33 ± 5	25 ± 6	23 ± 3

Table S3 Economic cost of this pathway in the study.

Economic cost	Parameters	Value	Unit	Unit price		Cost
Raw material cost	Nutrient medium	0.25	kg	400	CNY per kg	100
Consumable cost	10% Glycerol	1	kg	4.5	CNY per kg	4.5
Utilities cost	Water	0.345	kg	15	CNY per t	0.005175
	Electricity	0.7865	kWh	0.65	CNY per kWh	0.511225
Total cost						105.0164

Table S4 Engineered yeast strains and plasmids used in this study.

Strain name	Plasmid(s)	Pigment / Function
Yeast[CBD-GPI]	pCCW12-CBD	Anchoring module
Yeast[Mel1]	pCCW12-CBD, PTDH3-Mel1	Blue
Yeast[BLA]	pCCW12-CBD, PTDH3-BLA	Red
Yeast[Carotene]	pCCW12-CBD, pTDH3-CrtI, pTEF1-CrtE, pTEF2-tHGM1, pREV1-CrtYB	Orange
Yeast[Violacein]	pCCW12-CBD, pTDH3-VioA, pTEF1-VioB-VioE, pTEF2-VioC, pREV1-VioD	Purple

Table S5 Proteins and functional modules used in this study.

Type	Protein	Description
Enzyme	**Mel1**	α-galactosidase used for chromogenic substrate conversion to produce blue coloration
	**BLA**	β-lactamase used for chromogenic substrate conversion to produce red coloration.
	**VioA**	Violacein synthesis-related enzyme
	**VioB**	Violacein synthesis-related enzyme
	**VioC**	Violacein synthesis-related enzyme
	**VioD**	Violacein synthesis-related enzyme
	**VioE**	Violacein synthesis-related enzyme
	**CrtI**	Carotene synthesis-related enzyme
	**CrtE**	Carotene synthesis-related enzyme
	**CrtYB**	Carotene synthesis-related enzyme
	**tHGM1**	Carotene synthesis-related enzyme
Anchor proteins	**649 stalk-GPI**	Surface-anchoring protein for yeast display
Functional domain	**CBD**	A chitin-binding domain
promoter	**pTDH3**	Yeast constitutive promoters
	**pREV1**	
	**pTEF1**	
	**pCCW12**	
	**pTEF2**	
